# Stigma against people living with Human Immunodeficiency Virus: A quasi-experimental evaluation of active stigma reduction workshops among medical students in Tunisia

**DOI:** 10.1371/journal.pone.0350810

**Published:** 2026-06-11

**Authors:** Imen Mlouki, Emna Baoueb, Emna Hariz, Aya Ajmi Blout, Haythem Ayachi, Ahmed Moustafa, Sana El Mhamdi

**Affiliations:** 1 Preventive and Community Medicine, Faculty of Medicine of Monastir, University of Monastir, Monastir, Tunisia; 2 Department of Preventive and Community Medicine, Taher Sfar University Hospital, Mahdia, Tunisia; 3 Research Laboratory "Epidemiology Applied to Maternal and Child Health" 12SP17, Monastir, Tunisia; 4 Department of Human Anatomy and Physiology, Faculty of Health Sciences, University of Johannesburg, Johannesburg, South Africa; 5 Center for Data Analytics and School of Psychology, Bond University, Gold Coast, QLD, Australia; University of Zimbabwe Faculty of Medicine: University of Zimbabwe College of Health Sciences, ZIMBABWE

## Abstract

**Introduction:**

Stigma against people living with Human Immunodeficiency Virus (PLHIV), particularly from healthcare providers, continues to be a significant barrier discouraging individuals from seeking necessary care. However, HIV stigma, particularly among healthcare workers, is still underexplored, with limited focus on medical students. This study assesses the impact of active stigma reduction workshops on stigma towards PLHIV among a sample of medical students in Tunisia and identifies its related factors.

**Method:**

We performed a quasi-experimental study among all fifth-year students during the academic years 2024 and 2025 in the Faculty of Medicine of Monastir (Tunisia). We evaluated HIV stigma before and after each workshop via self-administrated and anonymous questionnaire. We used the validated versions of the brief HIV knowledge questionnaire and the Health Care Provider HIV/AIDS Stigma Scale.

**Results:**

A total of 216 medical students completely answered the questionnaire. Overall, most participants demonstrated a good level of HIV-related knowledge (88.4%). Good HIV knowledge, higher parental education and involvement in community or associative activities were significantly associated with lower stigma levels. Interestingly, using multivariate analyses, poor parental educational level and being stigmatized were independently associated with higher prejudice scores. This student-centered learning approach was revealed to be effective in significantly improving HIV-related knowledge and reducing HIV stigma among medical students.

**Conclusion:**

Our findings emphasize the value of integrating active learning methods into the medical curriculum to prepare future healthcare providers to deliver stigma-free care in order to promote ethical and inclusive behavior toward PLHIV.

## Introduction

The Human Immunodeficiency Virus (HIV) continues to be a significant global public health challenge. In 2023, it was reported that approximately 630,000 people died from HIV-related illness worldwide [1]. This disease has a significant economic effect which includes direct medical costs, lost productivity, and public health expenditures. In fact, according to the joint United Nations Program on HIV/AIDS (UNAIDS), the annual global cost of HIV/AIDS is estimated to be over $30 billion [2]. Despite major advancements in treatment and prevention, HIV continues to be a major health challenge especially in low and middle-income countries (LMIC). A study published in 2023 revealed an increasing rate in HIV incidence, mortality, and disability-adjusted life years across the Middle East and North Africa (MENA) region [3]. In Tunisia, the adult HIV prevalence is estimated at 0.1%, with approximately 8,000 people living with the virus in 2022 [4].

Several factors are associated with challenges in HIV care, including limited health education, socioeconomic disparities, inadequate health literacy and persistent knowledge gap [5,6]. Stigma and discrimination against people living with HIV (PLHIV) particularly from healthcare providers continues to be a significant barrier discouraging individuals from seeking necessary care [7,8]. According to a qualitative study conducted in Tunisia [9], healthcare provider misconceptions about this disease are one of the major leading causes to stigmatization and thus hinder combatting the HIV epidemic. In fact, HIV discrimination manifests as reluctance to provide care to PLHIV and use of extra precautionary measures resulting in delayed diagnosis and poor treatment adherence [10]. Sexual education remains a taboo and HIV infection is wrongly seen as a divine punishment and a consequence of immoral behavior especially in MENA countries [11]. This knowledge gap fuels stigma both among the general population and healthcare professionals [12,13]. However, HIV stigma is still underexplored in developing countries compared to developed ones, particularly among healthcare workers. For instance, a 2021 study in Sana’a, Yemen revealed discriminatory attitudes among healthcare workers stemming from a lack of institutional policies against HIV stigma [14,15].

Hence, educating healthcare providers is crucial in assessing their knowledge gap and reducing stigmatizing practices. Several studies have shown that the impact of training healthcare professionals on HIV transmission, prevention and treatment can improve their attitudes and reduce stigma [16]. According to a recent study conducted among healthcare workers in Vietnam [17], focused education programs raised engagement in HIV care and decreased stigma. It highlights the importance of educational trainings in improving healthcare worker knowledge and attitudes toward PLHIV. Addressing this issue among medical students in the medical curriculum is fundamental for establishing a generation of future healthcare professionals who can provide equitable and compassionate care to PLHIV. Indeed, medical student attitudes and knowledge about HIV are shaped during their training, making this population a key target for interventions aimed at reducing stigma [18]. However, most research on HIV-related stigma was among practicing clinicians, with limited focus on medical students, particularly in LMICs. A recent study in Saudi Arabia assessed this gap, reporting misconceptions and poor HIV knowledge among medical students. Thus, it is important to establish educational interventions among pre-clinical medical students.

Furthermore, while educational interventions have shown significant effects in reducing stigma, the effectiveness of active learning methods remains underexplored in the context of HIV [19]. For instance, a thematic review revealed that the student-centered learning (SCL) method has the ability to foster deeper engagement, empathy and understanding by placing learners at the center of the educational process preparing them for professional challenges [20].

Given these data, the current study aimed to assess the impact of active training (workshops) on reducing stigma against PLHIV among fifth medical students in the Faculty of Medicine of Monastir (Tunisia) and to explore its related factors.

## Method

### Sampling and data collection

We performed a quasi-experimental study during the academic years 2023–2024 and 2024–2025 among all fifth-year students in the Faculty of Medicine of Monastir (Tunisia), who have one remaining year of clinical training at the hospital during which they are responsible for patient care. The recruitment period for the first academic year was from 15/09/2023 to 31/05/2024, and for the second academic year from 15/09/2024 to 31/05/2025. The total duration of teaching during the academic year was 10 months.

A total of 216 students participated in our study, following the precedent set by a similar study by Al-Fadhli et al. [21]. Their survey assessed the impact of HIV stigma-reduction workshops on healthcare providers in two tertiary-care hospitals and three healthcare colleges in central India, including 650 participants, of whom 216 were medical students [21]. Despite different contexts, the intervention and population are similar, making it a relevant sample size reference.

Every week, a 2-hour workshop about HIV knowledge and practices was conducted for groups of 10 medical students at the Department of Preventive and Community Medicine within the faculty of Medicine of Monastir. The teacher, a professor of medicine, facilitated the sessions based on presentations and interactive lectures.

The intervention consisted of animating these workshops based on the SCL approach [20]. This method shifts the traditional focus from instructor-led teaching to one that places students at the heart of the learning experience. It focuses on courses and classroom-based role-plays lectures, and open discussions prepared and delivered actively by students.

We evaluated HIV knowledge and practices as well as stigma attitudes towards PLHIV before and after each workshop using self-administered and validated measurement tools.

### Measurement tools

The study instrument in the current study was a self-administered questionnaire, and consisted of three sections as follows:

#### • Knowledge about HIV among medical students.

To assess HIV knowledge, we used the validated French version of the **brief HIV knowledge questionnaire** (HIV-KQ-18). This questionnaire was originally developed by Carey et al. (2004) and was later validated in French [22]. This tool is self-administered, which is designed to assess general knowledge about HIV among medical students, modes of transmission, prevention methods and common misconceptions of the disease. It includes 18 yes or no statements evaluating attitudes and practices. Only five of the items are true (items 1, 4, 11, 14 and 17). The correct response is coded one, whereas the incorrect answers receive zero points with a maximum score obtainable for knowledge being 18 marks. Having a score of 14 or higher indicates a good HIV knowledge level, whereas a score below 14 signifies poor knowledge [23].

#### • Stigma attitudes towards PLHIV among medical students.

To evaluate stigma attitudes towards PLHIV, we used the validated instrument “**Health Care Provider HIV/AIDS Stigma Scale** (HPASS)” [24]. The HPASS consists of a 30-item structured questionnaire. Each item is an example of a scenario that physicians might encounter in their future professional life or certain beliefs regarding PLHIV. In each statement, responders choose from six possible answers ‘’ strongly disagree, ‘’ ‘’slightly disagree ‘’ disagree” ‘’agree,” ‘’slightly agree” or ‘’ strongly agree.” These responses are assigned corresponding scores of 0, 1, 2, 3, 4, 5, respectively. This tool evaluates three dimensions [24]: prejudice, stereotyping and discrimination, divided as follows:

- **Prejudice** (Items 1–13): These items reflect emotional reactions and negative attitudes towards PLHIV, including feelings of fear, blame or discomfort. A high score in this domain represents a stronger prejudicial attitude.
- **Stereotyping** (Items 14–23): These items evaluate negative beliefs towards PLHIV; assumptions about their behavior, lifestyle, or character. Stereotyping reflects cognitive biases often rooted in misinformation or cultural stigma
- **Discrimination** (Items 24–30): These items refer to discriminatory behavior or intentions, such as avoidance or unequal treatment of PLHIV. High scores indicate a greater likelihood of engaging in discriminatory actions.

In this section, we did not use predefined cut-off values to categorize participants into “low” or “high” stigma groups. Instead, the total score presented by the cumulative level of stigma, was considered as a continuous measure, with higher scores reflecting greater levels of stigma whether in the form of prejudice, stereotyping, or discriminatory behaviors. Comparing the mean scores before and after the intervention allows us to identify patterns or shifts in stigma and thus evaluate the impact of the active learning method [25].

#### • Sociodemographic characteristics and sexual practices of participants.

We collected data about sociodemographic characteristics and sexual practices to assess the potential influence of these factors on HIV related stigma. These questions were added based on a literature review about factors associated with HIV stigma. In the sociodemographic characteristics section, the collected data comprises: age, gender and parental educational level. Parental educational level was recorded into two categories: High educational level (at least one parent has completed higher education) and poor educational level (both parents have education levels below high school). We also asked about involvement in associative activities, HIV unit or screening campaigns and having received HIV-related training. The source of information on sexual and reproductive practices was grouped into reliable sources (Parents, Siblings, Cousins, School/University) and unreliable sources (Friends, Media, Internet). Data about experiencing stigma and sexual practices including: Previous sexual experience, condom protection, substance use during sex and sexually transmitted infections (STI) screening were collected.

**Cultural context:** Tunisia is a Muslim-majority country, and topics such as sexual behavior and HIV are often socially sensitive or somewhat taboo. In this context, HIV is generally associated with high-risk sexual practices, which may influence participants’ willingness to report certain behaviors or attitudes honestly. This consideration was taken into account when designing the questionnaire and interpreting the results.

### Statistics analysis

Data entry and analysis were conducted using the Statistical Package for social science (SPSS); Version 23.0. Qualitative variables were represented by effectives and percentages. After normal distribution testing, continuous variables were expressed as the mean plus or minus the standard deviation (mean ± SD).

To compare variables with HIV knowledge levels, we used the Chi 2 test (χ2) or Fisher exact test since we compared percentages. To determine factors associated with stigma score and its three dimensions, we performed a univariate analysis using the student test to compare two independent means. The multivariate analysis via the linear regression model was conducted to identify independent factors associated with stigma scores.

Regarding the evaluation of the impact of the active workshop, dependent sample t-tests were performed to compare the mean scores of HIV knowledge and stigma before and after the active training method at the significant level of 0.05. Also, we calculated the Relative Reduction (Percentage Change): as the difference between post-intervention and pre-intervention means, divided by the pre-intervention mean, and multiplied by 100.

### Ethical considerations

Participation in our study was voluntary. All participants provided informed consent and the study was anonymous. Students responded to the questionnaire in separate and distant tables. The data obtained from the students was solely used for the study purpose and was not included in the final score of the exam. The authors had no access to information that could identify students after data collection. The study protocol was originally submitted to the institutional ethics committee in 2023. Following a review of the protocol, the committee granted oral approval and an agreement in principle to proceed while formal documentation was being processed. Consequently, participant recruitment commenced in September 2023 under this initial oversight. The official written ethics approval was subsequently issued on February 8, 2024, by the Ethic Committee of the University Hospital in Mahdia (CEM 2024-02-08).

## Results

### Sociodemographic characteristics and sexual practices of participants

A total of 216 medical students answered the questionnaire completely with a response rate of 43.2%. The mean age of participants was 23 years±0.84 with a majority of female (63%). Participant background demographics and practices are outlined in Table 1.

**Table 1:** Sociodemographic characteristics and sexual practices among medical students.

Variables	Categories	Effective	Percentage
**Educational level of parents (N = 216)**	High	157	72.7%
	Poor	59	27.3%
**Involvement in associative activities, HIV unit** **or screening campaigns (N=216)**	Yes	50	23.1%
	No	166	76.9%
**Source of sexual knowledge (N = 216)**	Reliable	81	37.5%
	Unreliable	135	62.5%
**Experiencing stigma or discrimination (N = 216)**	Yes	31	14.4%
	No	185	85.6%
**Previous sexual experience (N = 216)**	Yes	28	13%
	No	188	87%
**Condom protection (N = 28)**	Yes	16	57.1%
	No	12	42.9%
**Substance use during sex (N = 28)**	Yes	24	50%
	No	24	50%
**STI screening (N = 216)**	Yes	5	2.3%
	No	211	97.7%

### Pre-intervention HIV stigma among medical students and its related factors

The majority of participants presented good knowledge levels (88.4%). Table 2 presents the results of univariate analysis on variables influencing the total stigma score among medical students. In fact, individuals having good HIV knowledge, high parental educational level and those who were involved in associative activities, HIV units or screening campaigns exhibited lower stigma scores (Table 2).

**Table 2:** Factors influencing the total stigma score among medical students: Univariate analysis.

Variables	Categories	Mean stigma score±SD	P value
**HIV knowledge (N = 216)**	Good	**59.34 ± 22.2**	**0.01**
	Poor	**69.88 ± 17.63**	
**Gender (N = 216)**	Male	59.76 ± 24.65	0.685
	Female	61.02 ± 20.31	
**Educational level of parents (N = 216)**	High	**58.65 ± 22.36**	**0.037**
	Poor	**65.64 ± 20.12**	
**Involvement in associative life** **HIV unit or screening campaigns (N = 216)**	Yes	**53.14 ± 22.07**	**0.006**
	No	**62.79 ± 21.47**	
**Source of sexual knowledge (N = 216)**	Reliable	60.26 ± 23.67	0.876
	Unreliable	60.74 ± 20.93	
**Experiencing stigma or discrimination (N = 216)**	Yes	64.48 ± 24.11	0.283
	No	60 ± 21.56	
**Previous Sexual experience (N = 216)**	Yes	61.43 ± 21.04	0.213
	No	54.67 ± 26.96	
**Condom protection (N = 28)**	Yes	46.56 ± 25.96	0.065
	No	65.5 ± 25.33	
**Substance use during sex (N = 28)**	Yes	55.54 ± 28.17	0.576
	No	59.46 ± 19.2	
**STI screening (N = 216)**	Yes	50.4 ± 20.41	0.296
	No	60.8 ± 22	

### Prejudice related factors

Table 3 highlights variables influencing prejudice among dimension among medical students through univariate analysis. Having a high educational level of parents and being involved in associative activities, HIV units or screening campaigns were associated with lower prejudice scores (Table 3).

**Table 3:** Factors influencing prejudice dimension among medical students: Univariate analysis.

Variables	Categories	Mean prejudice score±SD	P value
**HIV knowledge (N = 216)**	Good	26.68 ± 11.47	0.094
	Poor	30.72 ± 9.78	
**Gender (N = 216)**	Male	26.44 ± 12.684	0.489
	Female	27.55 ± 10.51	
**Educational level of parents (N = 216)**	High	**25.97 ± 11.25**	**0.013**
	Poor	**30.27 ± 11.07**	
**Involvement in associative activities,** **HIV unit or screening campaigns (N = 216)**	Yes	**22.7 ± 11.23**	**0.001**
	No	**28.49 ± 11.06**	
**Source of sexual knowledge(N = 216)**	Reliable	26.65 ± 12.43	0.621
	Unreliable	27.44 ± 10.67	
**Experiencing stigma or discrimination (N = 216)**	yes	**30.93 ± 13.74**	**0.044**
	No	**26.51 ± 10.8**	
**Previous Sexual experience (N = 216)**	Yes	25.28 ± 13.66	0.353
	No	27.42 ± 10.97	
**Condom protection (N = 28)**	Yes	21.93 ± 11.9	0.137
	No	29.75 ± 15.06	
**Substance use during sex (N = 28)**	Yes	26.04 ± 13.86	0.972
	No	26.16 ± 10.15	
**STI screening (N = 216)**	Yes	25.2 ± 10.1	0.699
	No	27.19 ± 11.38	

Multivariate linear regression showed that lower parental educational level and experiences of stigma or discrimination were independently associated with higher HIV prejudice scores (for more details, see Table 4).

**Table 4:** Factors influencing prejudice among medical students: Multivariate linear regression analysis.

Related Factors to HIV prejudice	Standard Errors	P value	B coefficient	IC95%
Educational level of parents (N = 216)	5.033	0.045	10.663	0.27 - 21.05
Experiencing stigma or discrimination (N = 216)	5.925	0.042	12.756	0.53 - 24.98

### Stereotype related factors

Table 5 shows variables influencing stereotype among medical students through univariate analysis.

**Table 5:** Factors influencing stereotype among medical students: Univariate analysis.

Variables	Categories	Mean stereotype score±SD	P value
**HIV knowledge (N = 216)**	Good	21.79 ± 8.15	0.166
	Poor	24.2 ± 8	
**Educational level of parents (N = 216)**	High	**21.34 ± 8.2**	**0.033**
	Poor	**24 ± 7.8**	
**Involvement in associative life** **HIV unit or screening campaigns (N = 216)**	Yes	22.54 ± 8.01	0.121
	No	20.5 ± 8.52	
**Previous Sexual experience (N = 216)**	Yes	19.67 ± 10.1	0.097
	No	22.42 ± 7.8	
**Condom protection (N = 28)**	Yes	**16.31 ± 10.34**	**0.039**
	No	**24.16 ± 8.13**	

### Discrimination dimension

No variables influencing the discrimination dimension among medical students were found through univariate analysis in our sample.

### Impact of SCL method on HIV knowledge and stigma against PLHIV

Table 6 shows the score trends before and after the active workshops (knowledge, stigma, prejudice, stereotype and discrimination scores).

**Table 6:** Impact of SCL method on HIV knowledge and stigma dimensions (N = 216).

Variable	Mean knowledge and stigma scores ±SD		P value	Relative change (%)
	Before	After		
**Knowledge score**	**15.57 ± 1.61**	**16.3 ± 1.5**	**<0.001**	**+4.69**
**Total Stigma score**	**60.56 ± 21.94**	**52.64 ± 22**	**<0.001**	**−13.08**
**Prejudice score**	**27.14 ± 11.34**	**22.57 ± 11.09**	**<0.001**	**−16.84**
**Stereotype score**	**22.07 ± 8.16**	**20.45 ± 8.51**	**<0.001**	**−7.34**
**Discrimination score**	**11.34 ± 7.54**	**9.61 ± 7.29**	**<0.001**	**−15.26**

Regarding HIV knowledge, the overall score increased significantly from 15.57 ± 1.61 to 16.3 ± 1.5. A notable reduction was observed in the stigma score following the SCL method. In fact, the mean score significantly decreased from 60.56 ± 21.94 to 52.64 ± 22. Similar results were found for all stigma dimensions (Table 6).

## Discussion

To our knowledge, our study is among the first in North Africa to focus on stigma among medical students against PLHIV and to assess the impact of the SCL method on both knowledge and stigma. In fact, we found that of all stigma dimensions, prejudice showed significant associations in multivariate analysis. Interestingly, poor parental educational level and being stigmatized were independently associated with higher prejudice scores. In addition, we found that the SCL method was effective in significantly improving HIV-related knowledge as well as in reducing stigma scores among medical students.

We found that the majority of participants were not involved in HIV related activities or screening campaigns (76.9%). This result is in contrast to findings in Hong Kong showing that 72.1% of final-year medical students were exposed to HIV patients during attachment to an HIV clinic in the late 2000s, compared with 39.3% in the mid-2010s [26]. The significantly lower exposure in Tunisia (23.1%) compared to Hong Kong likely stems from structural and institutional differences. Tunisian HIV care remains highly centralized in a few specialized units. Furthermore, pervasive socio-cultural stigma in Tunisia often necessitates a higher degree of patient anonymity, which, combined with strict confidentiality protocols, limits the integration of HIV-positive patients into general undergraduate clinical training. More broadly, in MENA countries, stigma, cultural taboos and lack of structured programs may contribute to the low involvement rates [11]. In our study, most medical students reported having an unreliable source of sexual knowledge (85.5%). However, studies in Iran and Greece reported greater use of reliable source [27]. This may be influenced by cultural and societal differences. In fact, in Tunisia, sexual health topics are still taboos leading younger individuals to seek false information elsewhere.

Among students who were sexually active in our sample, only 57.1% use condoms for protection. This contrasts with results among Mexican medical students in 2024, where a higher proportion of students reported condom use during vaginal intercourse (83.6% in men and 67.7% in women) [28]. However, condom use cannot be attributed only to the level of sexual health education. Indeed, other influencing factors such as partner preference, access to condoms, personal and cultural attitudes affect the use of this protective measure [29]. We found that only 2.3% of participants underwent STI screening. Similarly, in a 2023 study conducted in Warsaw, Poland, Only 21.43% of participants had ever been tested for HIV [30]. Several explanations could be cited such as low sexual encounters, fear of being stigmatized for testing and lack of HIV related knowledge [30]. In our cultural context, sexual health remains a sensitive topic, which may explain the very low STI screening rates and reliance on unreliable sources of information. These findings highlight the urgent need for broader, culturally appropriate sexual health education among medical students.

As for HIV knowledge, findings in our study showed a good knowledge level among 88.4% of medical students. In fact, in the Tunisian medical curriculum, students start learning about HIV and its impact early in their education. This is in accordance with results reported in Iran and in countries from both sub-Saharan Africa and North Africa [31,32]. Nevertheless, it remains essential to sustain efforts aimed at further enhancing HIV knowledge by screening the related factors and by developing targeted interventions in educational medical trainings. To the best of our knowledge, our study is the first to focus on stigmatizing attitudes towards PLHIV among medical students in Tunisia. As medical students play a key role in future care delivery, it is crucial to assess stigma and its related factors. Students with higher scores on HIV-related knowledge exhibit lower stigma rates. These results align with studies conducted in Yemen and Indonesia among healthcare professionals, where poor knowledge of HIV was linked to higher rates of stigma [14,33]. However, an Indonesian study suggested that physicians exhibited both the highest HIV knowledge and the highest levels of stigma [34]. Thus, combatting stigma requires interventions that transcend knowledge and highlight cultural beliefs and other factors influencing healthcare professionals’ attitudes. We found that female participants exhibit a higher stigma score compared to males. These results align with findings from an Iranian study [35] and contradicts with another study from the USA [36]. However, the gender difference observed in our study was not statistically significant and should therefore be interpreted with caution. In certain sociocultural contexts where discussions surrounding sexuality and HIV may be more socially constrained for women, gender-related norms could potentially influence attitudes. This hypothesis warrants further investigation in larger and more diverse samples. In our survey, students who were involved in associative activities, HIV units and screening campaigns exhibited a lower stigma score. Likewise, a study conducted in Jordan among medical students stated that pre-clinical students had a significantly more negative attitude towards PLHIV compared to their clinical counterparts [37]. Likewise, from a study carried out among students from Hong Kong, it was found that pupils who were not willing to provide care for PLHIV had less clinical experience with HIV patients [26]. We found that individuals untested for HIV tend to have a higher stigma score. These findings align with results from Indonesia [33]. One possible explanation is that during HIV testing and counseling, individuals might gain great HIV knowledge and undergo the emotional experience of diagnosis. Our study found no significant association between the reliability of the source and stigma rates. However, although the effect is weak, the media was found to be an enhancing factor for HIV knowledge and a reducing factor of HIV stigma in Iran [38]. The media is a powerful tool for combatting stigma against PLHIV when it is guided by accurate information about the disease. However, in many conservative societies, PLHIV continue to face negative representations in the media, reinforcing panic and misconceptions. Thus, it is urgent to implement media-based awareness and evidence-based education [39].

HIV prejudice refers to negative feelings, judgments, and attitudes towards PLHIV rooted in negative beliefs and often reinforced by fear of the disease [24]. We found that students with better HIV-related knowledge showed lower levels of prejudice. Similarly, dental healthcare providers in Pakistan with limited HIV-related knowledge tended to hold more negative attitudes towards HIV [40]. Participation in HIV-related workshops or screening campaigns was linked to a lower prejudice score in our sample. Likewise, trainings on HIV stigma and/or discrimination was associated with less negative attitudes in healthcare providers in Germany [41]. This highlights the importance of structured educational interventions for reducing prejudice among healthcare professionals. Beyond the univariate analysis, multivariate analysis of stigma dimensions revealed specific predictors of prejudice. Notably, previous personal experience of being stigmatized and poor parental educational level were independently associated with higher prejudice scores. Limited research has explored how an individual's own history of being stigmatized shapes their attitudes toward PLHIV. However, based on results reported in Zambia, healthcare professionals who did not stigmatize but witnessed stigmatizing attitudes among their coworkers, were more likely to hold critical and stigmatizing attitudes [42]. We did not find evidence of an association between the parental educational level of students and their attitude towards PLHIV. On the other hand, in Indonesia, belonging to the richest or rich economic group was associated with higher levels of stigma against PLHIV [43]. In contrast, a Ugandan study reported that wealthier individuals were less likely to endorse social distance and stigma against PLHIV [44]. These findings suggest that neither wealth nor parental education level predicts young people's attitudes. To the best of our knowledge, this is the first study to report associated factors with prejudice scores. Nonetheless, factors such as relationship dynamics, personal convictions, and social barriers must also be taken into account when evaluating attitudes toward HIV. Assessing related factors to HIV stigma into medical curriculum is needed to prepare future professionals into delivering compassionate and equitable care to PLHIV and to combat HIV disease [45].

Reducing stigma is essential to improving healthcare outcomes and care delivery especially among healthcare professionals [17,46]. Through the years, Depersonalization of patient cases and lack of diverse learning methods have limited student engagement and empathy. Traditional learning approaches often fail to nurture humanistic and relational skills. As a result, various learning methods have been used in different curricula and medical education such as problem-based learning, Case-based discussions, and flipped-classroom methods. Another innovative approaches involve Game-based learning, Team-based learning, Simulation-based learning, and peer teaching. They all embody the concept of SCL placing the learner at the core of the educational process [47]. It involves active participation where students engage in role-playing scenarios, open discussions and lectures. Students take on the roles of actors in various role-playing scenarios and are often placed in patient and doctor shoes [48]. Further, a range of educational interventions has been employed for HIV-stigma reduction [16]. Thus, we aimed to assess the impact and effectiveness of this method in enhancing HIV-related knowledge and reducing stigma among medical students. In our study, after the SCL approach, the overall HIV knowledge score significantly improved. A significant decline in HIV stigma scores was also noted and similar improvements were observed across all stigma dimensions. For instance, similar Tunisian data showed that both active participants and observers achieved significant gains in knowledge scores after a role-play simulation [49]. Similarly, a Tunisian study assessed the impact of the SCL among medical students in the faculty of medicine of Monastir. Significant improvement in hand hygiene and biomedical waste management after the SCL intervention [50].

Beyond its implication in general medical subjects, the SCL method has also been explored in our primary focus, enhancing HIV-related knowledge and reducing stigma against PLHIV. Given the HPASS scoring range, the observed 7.9-point decrease reflects a tangible reduction in stigmatizing attitudes, indicating that the intervention may have practical educational relevance in shaping future healthcare professionals’ perspectives toward PLHIV. For instance, a peer-led education intervention among secondary school students in Sudan showed a significant improvement in participant knowledge, attitudes and practices regarding HIV. This study highlights the effectiveness of SCL and peer-based approaches in promoting HIV awareness and reducing HIV stigma among medical students [51]. Similarly, a pilot study in the United States assessed the Facilitated Resilience and Equity through Stigma-reduction and Health workshop, a reduction of HIV-related stigma intervention among healthcare workers. During the workshop, participants interacted through discussions and role-playing. Findings showed a significant reduction in stigma and a significant HIV-related knowledge increase [52]. In addition, in China, a web-based game was developed to reduce HIV-related stigma. The game used participatory gamification and storytelling to immerse university students in the lives of PLHIV. This approach enhanced empathy-building, active learning through problem solving and reflection [53]. In addition, our study results align with a previous Indian study conducted among healthcare professionals [21]. The HIV-related stigma reduction workshops in Central India significantly impacted the knowledge and attitudes of healthcare providers and students. These results were assessed through a pre and post-test similar to our intervention.

To our knowledge, our study is the first globally and in the MENA region to simultaneously integrate the HIV-KQ-18 and the HPASS in a single research framework among medical students. HIV knowledge and stigma are critical issues in the MENA region, where persistent misconceptions, cultural taboos, and deeply rooted stigma continue to hinder efforts in HIV prevention and patient care. Addressing these challenges among medical students as future healthcare providers is an essential urgent step toward a long-term stigma-free environment and improved quality of life for PLHIV. It is essential to form medical graduates with a solid foundation in professional ethics, empathy, and a clear understanding of patient rights and confidentiality, ensuring they are prepared to deliver respectful and stigma-free care for patients. Uniquely, our study also incorporates a before-and-after intervention design, embedding active training and SCL modules within the medical education curriculum.

These results should be considered in light of the study limitations. First, it was a single-center study with a limited sample size. Since the study was conducted among medical students from one institute, findings cannot be generalized to broader populations of medical or healthcare students. Similar constraints were observed in a Vietnamese study among medical students who assessed HIV knowledge and stigma among a limited group of 200 medical students [54]. In addition, the Faculty of Medicine of Monastir operates within a specific educational, institutional, and sociocultural context that may influence students’ attitudes toward PLHIV. Tunisia is part of the MENA region, where HIV-related stigma is often shaped by religious norms, cultural perceptions surrounding sexuality, and societal attitudes toward key populations. These contextual factors may influence baseline stigma levels as well as responsiveness to educational interventions. Furthermore, variations in curriculum content, clinical exposure to PLHIV, and institutional culture across medical faculties may affect the transferability of our findings to other settings, both nationally and internationally. To enhance the generalizability and reliability of the findings, it is recommended to generalize our intervention to all healthcare institutes on a national scale for reliable results. Secondly, one important limitation of this study was the lack of future follow-up of the impact of the intervention. While the training aimed to reduce HIV stigmatizing attitudes among medical students, changes in practices among healthcare professionals were not evaluated. The current research did not conduct long-term follow-up surveys to verify the sustainability of the training, making it impossible to determine whether the training truly had a positive impact on the participant behavior against PLHIV. This gap between theoretical training and healthcare practices is highlighted in an Indian study, which emphasized the absence of stage-based follow-up to assess the effectiveness of the reduction workshops [21]. Future studies should integrate multiple follow-up stages for a deeper understanding of how knowledge and attitudes evolve and potentially translate into lasting behavioral change. Furthermore, because stigma was measured immediately after the intervention, we cannot determine whether the observed reduction reflects a sustained change in attitudes or a short-term response. Future research is planned to include longitudinal follow-up assessments at multiple time points to evaluate the durability of attitude change over time. Thirdly, in our study, the relatively low response rate (43.2%) may reflect potential bias, as some students may have chosen not to participate due to the sensitive nature of certain questions related to sexuality and religious or cultural norms.

## Conclusion

Our findings underscore the urgent need to integrate continuous anti-stigma education into medical curricula through active learning approaches. Interventions must be introduced early in the medical curriculum and backed by strong institutional commitment. Moreover, such efforts must be sustained and extend beyond healthcare settings to target the general population fostering an informed and nondiscriminatory society. Within the healthcare environment and the general population, a graduated accountability system should be introduced to address stigma early on.

## Supporting information

S1 File Questionnaire used for pre- and post-intervention data collection. This file contains the questionnaire administered before and after the intervention.
(DOCX)

S2 File Anonymized Excel dataset (.csv). This file contains the data used to conduct the analyses presented in this study.
(CSV)
