# Supplementary material for: Stigma against people living with Human Immunodeficiency Virus: A quasi-experimental evaluation of active stigma reduction workshops among medical students in Tunisia
Source: PLoS One. 2026 Jun 11;21(6):e0350810. doi: 10.1371/journal.pone.0350810 (PMC13257959; doi:10.1371/journal.pone.0350810)
Supplement: S1 File — This file contains the questionnaire administered before and after the intervention. (DOCX) [file pone.0350810.s001.docx]

**Students’ Knowledge about HIV**

**Please answer the following questions to the best of your knowledge. Thank you.**

| **Statement** | **True** | **False** |
| --- | --- | --- |
| **1. Coughing or sneezing does not transmit HIV.** | **☐** | **☐** |
| **2. A person can contract HIV by sharing a drinking glass with someone who is infected with HIV.** | **☐** | **☐** |
| **3. During sexual intercourse, withdrawing the penis before ejaculation prevents a woman from contracting HIV.** | **☐** | **☐** |
| **4. A woman can contract HIV if she has anal sex with a man.** | **☐** | **☐** |
| **5. Taking a shower or washing one’s genitals after sexual intercourse prevents HIV infection.** | **☐** | **☐** |
| **6. All pregnant women infected with HIV will give birth to a child with AIDS.** | **☐** | **☐** |
| **7. People who are at risk of contracting HIV quickly show serious signs and symptoms of infection.** | **☐** | **☐** |
| **8. There is a vaccine that can prevent adults from contracting HIV.** | **☐** | **☐** |
| **9. People are at risk of contracting HIV when they engage in deep kissing / tongue kissing with a partner living with HIV.** | **☐** | **☐** |
| **10. A woman cannot contract HIV if she has sexual intercourse during menstruation.** | **☐** | **☐** |
| **11. There is a female condom that can reduce the chances of a woman contracting HIV.** | **☐** | **☐** |
| **12. A natural membrane condom is more effective than a latex condom in preventing HIV infection.** | **☐** | **☐** |
| **13. A person cannot contract HIV if they are taking antibiotics.** | **☐** | **☐** |
| **14. Having sexual intercourse with more than one partner can increase the chances of contracting HIV.** | **☐** | **☐** |
| **15. Taking an HIV test one week after sexual intercourse can detect whether a person is infected with HIV.** | **☐** | **☐** |
| **16. A person can contract HIV by being in a spa or swimming pool at the same time as someone infected with HIV.** | **☐** | **☐** |
| **17. A person can contract HIV through oral sex.** | **☐** | **☐** |
| **18. Using petroleum jelly (Vaseline) or baby oil with a condom reduces the chances of contracting HIV.** | **☐** | **☐** |

**Attitudes toward people living with HIV**

|  | Strongly disagree | Slightly disagree | Disagree | Agree | Slightly agree | Strongly agree |
| --- | --- | --- | --- | --- | --- | --- |
| 1. HIV patients make me uncomfortable. |  |  |  |  |  |  |
| 2. I worry about contracting the virus from HIV patients. |  |  |  |  |  |  |
| 3. I would rather not come into physical contact with HIV  Patients. |  |  |  |  |  |  |
| 4. HIV patients present a threat to my health. |  |  |  |  |  |  |
| 5. It is a little scary to think I have touched HIV patients. |  |  |  |  |  |  |
| 6. I worry that universal precautions are not good enough to  protect me from HIV patients. |  |  |  |  |  |  |
| 7. I would rather see an HIV-negative patient than see an HIV  patient with non-HIV-related concerns. |  |  |  |  |  |  |
| 8. HIV patients present a threat to the health of other patients. |  |  |  |  |  |  |
| 9. I would be hesitant to send HIV patients to get blood work  done due to my fear of others’ safety. |  |  |  |  |  |  |
| 10. I would feel uncomfortable knowing one of my colleagues is  HIV+. |  |  |  |  |  |  |
| 11. I would be comfortable working alongside another health  care provider who has HIV. |  |  |  |  |  |  |
| 12. I would want to wear two sets of gloves when examining  HIV patients. |  |  |  |  |  |  |
| 13. It would be hard to react calmly if a patient tells me he or  she is HIV+. |  |  |  |  |  |  |
| 14. HIV patients tend to have numerous sexual partners. |  |  |  |  |  |  |
| 15. HIV patients who have acquired HIV through sex are more at fault for contracting HIV than those who have acquired HIV  through a blood transfusion. |  |  |  |  |  |  |
| 16. I think HIV patients have engaged in risky activities despite  knowing these risks. |  |  |  |  |  |  |
| 17. I often think HIV patients have caused their own health. |  |  |  |  |  |  |
| 18. I think if people act responsibly, they will not contract HIV. |  |  |  |  |  |  |
| 19. I believe most HIV patients acquired the virus through risky  behavior. |  |  |  |  |  |  |
| 20. I think many HIV patients likely have substance abuse  problems. |  |  |  |  |  |  |
| 21. HIV patients should accept responsibility for acquiring the  virus. |  |  |  |  |  |  |
| 22. I tend to think that HIV patients do not share the same  values as me. |  |  |  |  |  |  |
| 23. I think people would not get HIV if they had sex with fewer  people. |  |  |  |  |  |  |
| 24. I believe I have the right to refuse to treat HIV patients: |  |  |  |  |  |  |
| 25. to protect myself. |  |  |  |  |  |  |
| 26. if I feel uncomfortable. |  |  |  |  |  |  |
| 27. if other staff members are concerned about safety. |  |  |  |  |  |  |
| 28. for the safety of other patients. |  |  |  |  |  |  |
| 29. if I am concerned about legal liability. |  |  |  |  |  |  |
| 30. I would avoid conducting certain procedures on HIV  patients. |  |  |  |  |  |  |

**Sexual Practices among Students**

1. Have you ever had sexual intercourse (oral / anal / vaginal)?
   ☐ Yes  ☐ No
2. Do you use condoms?
   ☐ Always ☐ Often ☐ Sometimes ☐ Rarely ☐ Never

If you do not use condoms, why?
☐ You do not know how to use them
☐ You are embarrassed to buy them
☐ You do not like them
☐ You trust your partner

1. Have you ever used psychoactive substances or alcohol during sexual intercourse?
   ☐ Yes ☐ No ☐ I am not sexually active
2. Have you been tested for sexually transmitted infections (STIs) during the past year?
   ☐ Yes ☐ No

**Participant Information**

- Age: ______
- Gender: ☐ Man ☐ Woman ☐ Other

**Parental education level:**

**Mother:** ☐ No formal education ☐ Primary ☐ Secondary ☐ University
**Father:** ☐ No formal education ☐ Primary ☐ Secondary ☐ University

- Are you active in community/associative life?
  ☐ Yes ☐ No
- Have you ever had exposure/rotation in a unit caring for HIV-positive patients?
  ☐ Yes ☐ No
- Have you participated in any training workshop on HIV or HIV screening campaigns?
  ☐ Yes ☐ No

**Source of information on sexual and reproductive health:**

☐ Parents
☐ Siblings
☐ Cousins
☐ Friends
☐ School/University
☐ Media (TV/Magazines/Films…)
☐ Internet

- Have you ever been a victim of stigmatization/discrimination for any reason?
  ☐ Yes ☐ No
