# Supplementary material for: Stigma against people living with Human Immunodeficiency Virus: A quasi-experimental evaluation of active stigma reduction workshops among medical students in Tunisia
Source: PLoS One. 2026 Jun 11;21(6):e0350810. doi: 10.1371/journal.pone.0350810 (PMC13257959; doi:10.1371/journal.pone.0350810)
Supplement: Attachment — Submitted filename: PONE-D-25-49448_R4_reviewer.pdf [file pone.0350810.s006.pdf]

PLOS One

Stigma against People Living with Human Immunodeficiency Virus: A quasi-experimental evaluation of Active Stigma Reduction Workshops among Medical Students in Tunisia

--Manuscript Draft--

**Manuscript Number:**	PONE-D-25-49448R1
**Article Type:**	Research Article
**Full Title:**	Stigma against People Living with Human Immunodeficiency Virus: A quasi-experimental evaluation of Active Stigma Reduction Workshops among Medical Students in Tunisia
**Short Title:**	Stigma Reduction Workshops on HIV: A Quasi-Experimental Study among Tunisian Medical Students
**Corresponding Author:**	Emna Hariz<br>Universite de Monastir Faculte de Medecine de Monastir<br>mahdia, TUNISIA
**Keywords:**	Stigma; HIV; Medical student; Education; Tunisia
**Abstract:**	Introduction<br><br>Stigma against people living with Human Immunodeficiency Virus (PLHIV), particularly from healthcare providers, continues to be a significant barrier discouraging individuals from seeking necessary care. However, HIV stigma, particularly among healthcare workers, is still underexplored, with limited focus on medical students. This study assesses the impact of active stigma reduction workshops on stigma towards PLHIV among a sample of medical student in Tunisia and identifies its related factors.<br><br>Method<br><br>We performed a quasi-experimental study among all fifth-year students during the academic years 2024 and 2025 in the Faculty of Medicine of Monastir (Tunisia). We evaluated HIV stigma before and after each workshop via self-administrated and anonymous questionnaire. We used the validated versions of the brief HIV knowledge questionnaire and the Health Care Provider HIV/AIDS Stigma Scale.<br><br>Results<br><br>A total of 216 medical students completely answered the questionnaire. Overall, most participants demonstrated a good level of HIV-related knowledge (88.4%). Good HIV knowledge, higher parental education and involvement in community or associative activities were significantly associated with lower stigma levels.Interestingly, using multivariate analyses, poor parental educational level and being stigmatized were independently associated with higher prejudice scores.This student-centered learning approach was revealed to be effective in significantly improving HIV-related knowledge and reducing HIV stigma among medical students.<br><br>Conclusion<br><br>Our findings emphasize the value of integrating active learning methods into medical curriculum to prepare future healthcare providers to deliver stigma-free care in order to promote ethical and inclusive behavior toward PLHIV.
**Order of Authors:**	Imen Mlouki
	Emna Baoueb
	Emna Hariz
	Aya Ajmi Blout
	Haythem Ayachi

Powered by Editorial Manager® and ProduXion Manager® from Aries Systems Corporation

	Ahmed Moustafa
	Sana El Mhamdi
**Response to Reviewers:**	Dear Dr. Adekola and Reviewers,<br>We sincerely thank you for your thoughtful and constructive comments. We appreciate the time and expertise invested in reviewing our manuscript. We have carefully revised the manuscript to address all concerns raised. Below, we provide a detailed, point-by-point response. All changes have been highlighted in the revised manuscript with track changes.<br>Emna Hariz<br><br>Response to Editorial Requirements<br>Formatting and File Naming<br>The manuscript has been carefully revised to comply fully with PLOS ONE formatting guidelines, including title page structure, section organization, reference formatting, and file naming conventions.<br>Supporting Information Captions<br>Captions for all Supporting Information files have been added at the end of the manuscript.<br>Supporting Information File (S1_Data.csv)<br>The Supporting Information file S1 has been re-saved. An Excel version has been uploaded.<br>Reference List Review<br>The reference list has been thoroughly reviewed for completeness and accuracy.<br>Response to Academic Editor<br>Two assumptions the author seem to make, and should revisit:<br>Comment 1: That statistical significance alone adequately demonstrates intervention value.<br>We thank the editor for this comment. We have carefully considered this point and addressed it in several places in the manuscript, following suggestions made by the reviewers, to ensure that we discuss both the statistical and practical significance of the intervention.<br>Comment 2: That readers will intuitively understand the local educational and cultural context.<br>We agree with the editor's observation and have already made revisions in the methods section to provide clearer explanations of the local educational and cultural context. (page 5, line184).<br>Response to reviewers:<br>Reviewer #1:<br>The manuscript is technically sound, methods well explained and statistical analysis conducted satisfactorily. The results presented support the conclusion as well.<br>Thank you for your positive feedback.<br>The manuscript needs few minor revisions, including grammar and spelling checks.<br>There are a few revisions necessary, as follows:<br>1.Please recheck the grammar and spelling throughout the manuscript.<br>We have carefully revised the manuscript to correct grammar.<br>2.In the Method section, please elaborate on the weekly workshop about HIV knowledge and practices- the trainer/teacher qualification, total duration of teaching (in weeks/ months), etc.<br>We thank the reviewer for this helpful suggestion. We have expanded the Methods section, including the trainer's qualifications (professor of medicine) and the total duration of teaching in one academic year (10 months) (page 3, lines 115 and 123).

3.Page 19, reference 7:- Please add author/organization/website name.
The reference has been revised to include the authoring organization and website name.
4.Please ensure the references are complete and in a standard format.
We have carefully reviewed and standardized all references to ensure completeness and accuracy.
Reviewer #2:
This manuscript addresses an important and underexplored issue: HIV-related stigma among medical students in Tunisia. The focus on the MENA region is timely, and the use of an active, student-centred workshop intervention is appropriate. The quasi-experimental pre–post design and use of validated instruments (HIV-KQ-18 and HPASS) are clear strengths. Overall, the study is sound, but several clarifications are needed to ensure proper interpretation.
We sincerely thank the reviewer for the positive evaluation of our work and for recognizing the relevance and methodological strengths of the study. We appreciate the constructive feedback provided.
First, baseline HIV knowledge is not clearly reported. The statement that 88.4% of participants had “good” knowledge is ambiguous, as it is unclear whether this reflects baseline or post-intervention scores. This must be clarified in the Methods and Results.
We thank the reviewer for highlighting this ambiguity. The 88.4% refers to baseline (pre-intervention) HIV knowledge. This has now been explicitly clarified in the revised manuscript (page 8, line 218).
A 7.9-point reduction in stigma scores (Table 5) is reported as statistically significant, but its practical or clinical relevance is not discussed. Readers need context regarding what constitutes a meaningful change on the HPASS scale.
Action: Discuss the practical significance of the observed stigma reduction and, where possible, compare it with findings from similar studies.
We thank the reviewer for this important comment. We have revised the Discussion section to address the practical significance of the 7.9-point reduction in HPASS scores. We have also contextualized our findings by comparing them with similar interventions in Sudan, the United States, China, and India (page 16, line 385).
All participants were recruited from a single medical faculty. While acknowledged, this limitation is not sufficiently discussed in relation to cultural, institutional, and educational context.
Action: Expand the discussion on how context-specific factors may limit generalizability beyond the Faculty of Medicine of Monastir.
We thank the reviewer for this observation. We have expanded the Discussion section to further elaborate on how sociocultural norms, religious influences, institutional culture, and variations in medical curricula may limit the generalizability of our findings beyond the Faculty of Medicine of Monastir (page 17, lines 418).
The study assesses stigma immediately post-intervention only. Without follow-up, the durability of attitude change cannot be evaluated.
Action: Explicitly acknowledge this limitation and outline plans for follow-up assessments in future studies.
We thank the reviewer for this comment. We have strengthened the Discussion section to explicitly state that stigma was assessed only immediately post-intervention and that the durability of attitude change cannot be determined. We have also added a statement outlining plans for future longitudinal follow-up assessments to evaluate the sustainability of the intervention effects (page 18, line 438).
The relatively low response rate raises concerns about potential selection bias.
Action: Discuss how respondents may differ from non-respondents and the implications for interpretation.
We thank the reviewer for this important comment. We have expanded the Limitations section to acknowledge that the relatively low response rate (43.2%) may introduce selection bias. (page 18, line 442).
The regression results are inconsistently reported (negative coefficient with a confidence interval crossing zero), making interpretation difficult.
Action: Provide a complete regression table including coefficients, confidence intervals, and standard errors for all predictors.
We thank the reviewer for this observation. We have revised the manuscript to include a complete regression table (Table 4) presenting coefficients, 95% confidence intervals, and standard errors.
Results rely primarily on p-values, which limits interpretability.
Action: Add effect size indicators or percentage changes (e.g., relative reduction in

Powered by Editorial Manager® and ProduXion Manager® from Aries Systems Corporation

	stigma scores).<br>We thank the reviewer for this suggestion. We have added the relative percentage changes in knowledge and stigma scores to the Results section to provide additional context regarding the magnitude of the observed effects (Table 6).<br>Female students reportedly showed higher stigma levels than males, but no explanation is provided.<br>Action: Discuss possible cultural or contextual mechanisms underlying this finding.<br>We thank the reviewer for this insightful comment. We added a discussion of potential sociocultural influences, including gender-related norms surrounding sexuality and HIV in certain contexts, which may shape attitudes. (page 14, line 315).<br>The very low STI screening rate (2.3%) and heavy reliance on unreliable sources for sexual health information have important implications.<br>Action: Expand discussion on how these findings inform the need for broader sexual health education.<br>We thank the reviewer for this comment. We have expanded the Discussion section to highlight that, in our cultural context, sexual health remains a sensitive topic, which may explain the very low STI screening rates and reliance on informal or unreliable sources. We emphasize that these findings underscore the urgent need for broader, culturally appropriate sexual health education among medical students (page 13, line 295).<br>Comparisons with other settings (e.g., Hong Kong) lack sufficient contextual explanation.<br>Action: Clarify institutional, cultural, or legal barriers limiting HIV-related clinical exposure in Tunisia.<br>We thank the reviewer for this comment. We have revised the Discussion section to clarify that institutional, cultural, and legal factors in Tunisia may limit medical students' clinical exposure to HIV patients (page 13, line 274)<br>There is an apparent discrepancy between the ethics approval date (February 2024) and the reported start of recruitment (September 2023).<br>Action: Clarify the ethics approval timeline.<br>We thank the reviewer for highlighting this important point and appreciate the opportunity to clarify. The study protocol was submitted to the institutional ethics committee in 2023, and we received oral approval to proceed while the formal documentation was pending due to procedural delays within the committee.<br>Recruitment only began once we had received this agreement in principle, ensuring that ethical considerations were respected. The official written ethics approval was subsequently issued in February 2024.<br>The current statement does not clearly meet PLOS ONE requirements for public data access.<br>Action: Specify the public repository and confirm unrestricted access.<br>We thank the reviewer for this comment. The Data Availability Statement has been revised to clearly indicate that all underlying data are fully available in the Supporting Information file, including the saved Excel dataset (S1_Data.csv), ensuring unrestricted access (page 19, line 458).<br>Reviewer #3:<br>This manuscript is well written. The abstract was a representative summary of the manuscript. The background provided sufficient clarity on the rationale and objective of the study. The methods were adequately described to enable reproducibility. The findings were well discoursed and situated in existing literature.<br>Your constructive appraisal is highly valued and motivates us to continue improving the quality and rigor of our work.
**Additional Information:**	
**Question**	**Response**
**Financial Disclosure**<br><br>Enter a financial disclosure statement that describes the sources of funding for the work included in this submission. Review the submission guidelines for detailed requirements. View published research articles from *PLOS ONE* for specific	The author(s) received no specific funding for this work.

Powered by Editorial Manager® and ProduXion Manager® from Aries Systems Corporation

examples.<br><br>This statement is required for submission and **will appear in the published article** if the submission is accepted. Please make sure it is accurate.<br><br>**Funded studies**<br>Enter a statement with the following details:<br>• Initials of the authors who received each award<br>• Grant numbers awarded to each author<br>• The full name of each funder<br>• URL of each funder website<br>• Did the sponsors or funders play any role in the study design, data collection and analysis, decision to publish, or preparation of the manuscript?<br><br>Did you receive funding for this work?	
**Competing Interests**<br><br>Use the instructions below to enter a competing interest statement for this submission. On behalf of all authors, disclose any competing interests that could be perceived to bias this work—acknowledging all financial support and any other relevant financial or non-financial competing interests.<br><br>This statement is required for submission and **will appear in the published article** if the submission is accepted. Please make sure it is accurate and that any funding sources listed in your Funding Information later in the submission form are also declared in your Financial Disclosure statement.<br><br>View published research articles from *PLOS ONE* for specific examples.	The authors have declared that no competing interests exist.

**NO authors have competing interests**<br><br>Enter: *The authors have declared that no competing interests exist.*<br><br>**Authors with competing interests**<br><br>Enter competing interest details beginning with this statement:<br><br>*I have read the journal's policy and the authors of this manuscript have the following competing interests: [insert competing interests here]*<br><br>* typeset	
**Ethics Statement**<br><br>Enter an ethics statement for this submission. This statement is required if the study involved:<br><br>• Human participants<br>• Human specimens or tissue<br>• Vertebrate animals or cephalopods<br>• Vertebrate embryos or tissues<br>• Field research<br><br>Write "N/A" if the submission does not require an ethics statement.<br><br>General guidance is provided below. Consult the submission guidelines for detailed instructions. **Make sure that all information entered here is included in the Methods section of the manuscript.**	Participation in our study was voluntary. All participants provided informed consent and the study was anonymous. Students responded to the questionnaire in separate and distant tables. The data obtained from the students was solely used for the study purpose and were not included in the final score of the exam. The Ethic Committee of the University Hospital in Mahdia (CEM 2024-02-08) approved the study protocol.

**Format for specific study types**<br><br>**Human Subject Research (involving human participants and/or tissue)**<br>• Give the name of the institutional review board or ethics committee that approved the study<br>• Include the approval number and/or a statement indicating approval of this research<br>• Indicate the form of consent obtained (written/oral) or the reason that consent was not obtained (e.g. the data were analyzed anonymously)<br><br>**Animal Research (involving vertebrate animals, embryos or tissues)**<br>• Provide the name of the Institutional Animal Care and Use Committee (IACUC) or other relevant ethics board that reviewed the study protocol, and indicate whether they approved this research or granted a formal waiver of ethical approval<br>• Include an approval number if one was obtained<br>• If the study involved *non-human primates*, add *additional details* about animal welfare and steps taken to ameliorate suffering<br>• If anesthesia, euthanasia, or any kind of animal sacrifice is part of the study, include briefly which substances and/or methods were applied<br><br>**Field Research**<br><br>Include the following details if this study involves the collection of plant, animal, or other materials from a natural setting:<br>• Field permit number<br>• Name of the institution or relevant body that granted permission	
**Data Availability**<br><br>Authors are required to make all data underlying the findings described fully available, without restriction, and from the time of publication. PLOS allows rare exceptions to address legal and ethical concerns. See the PLOS Data Policy and FAQ for detailed information.	Yes - all data are fully available without restriction

Powered by Editorial Manager® and ProduXion Manager® from Aries Systems Corporation

A Data Availability Statement describing where the data can be found is required at submission. Your answers to this question constitute the Data Availability Statement and **will be published in the article**, if accepted.<br><br>**Important:** Stating 'data available on request from the author' is not sufficient. If your data are only available upon request, select 'No' for the first question and explain your exceptional situation in the text box.<br><br>Do the authors confirm that all data underlying the findings described in their manuscript are fully available without restriction?	
**Describe where the data may be found in full sentences. If you are copying our sample text, replace any instances of XXX with the appropriate details.**<br><br>• If the data are **held or will be held in a public repository**, include URLs, accession numbers or DOIs. If this information will only be available after acceptance, indicate this by ticking the box below. For example: *All XXX files are available from the XXX database (accession number(s) XXX, XXX.*).<br>• If the data are all contained **within the manuscript and/or Supporting Information files**, enter the following: *All relevant data are within the manuscript and its Supporting Information files.*<br>• If neither of these applies but you are able to provide **details of access elsewhere**, with or without limitations, please do so. For example:<br><br>*Data cannot be shared publicly because of [XXX]. Data are available from the XXX Institutional Data Access / Ethics Committee (contact via XXX) for researchers who meet the criteria for access to confidential data.*<br><br>*The data underlying the results presented in the study are available from (include the name of the third party*	All relevant data are within the manuscript and its Supporting Information files.

and contact information or URL).<br>• This text is appropriate if the data are owned by a third party and authors do not have permission to share the data.<br><br>* typeset	
Additional data availability information:	

Manuscript Click here to access/download;Manuscript;Manuscript.docx

Stigma against People Living with Human Immunodeficiency Virus: A quasi-experimental evaluation of Active Stigma Reduction Workshops among Medical Students in Tunisia

Authors: Imen Mlouki[1,2,3], Emna Baoueb[1], Emna Hariz[2,3*], Aya Ajmi Blout[1,2], Haythem Ayachi[1], Ahmed Moustafa[4], Sana El Mhamdi[1,2,3].

1Preventive and Community Medicine, Faculty of Medicine of Monastir, University of Monastir, Monastir, Tunisia.

2Department of Preventive and Community Medicine, Taher Sfar University Hospital, Mahdia, Tunisia.

3Research Laboratory "Epidemiology Applied to Maternal and Child Health" 12SP17, Monastir, Tunisia.

4School of Psychology, Bond University, Australia

*Corresponding author: Emna Hariz

Department of Preventive and Community Medicine, Taher Sfar University Hospital, Mahdia, Tunisia, Research Laboratory "Epidemiology Applied to Maternal and Child Health" 12SP17, Monastir, Tunisia.

Email: emna.harizz@gmail.com

Abstract

Introduction: Stigma against people living with Human Immunodeficiency Virus (PLHIV), particularly from healthcare providers, continues to be a significant barrier discouraging individuals from seeking necessary care. However, HIV stigma, particularly among healthcare workers, is still underexplored, with limited focus on medical students. This study assesses the impact of active stigma reduction workshops on stigma towards PLHIV among a sample of medical students in Tunisia and identifies its related factors.

Method: We performed a quasi-experimental study among all fifth-year students during the academic years 2024 and 2025 in the Faculty of Medicine of Monastir (Tunisia). We evaluated HIV stigma before and after each workshop via self-administrated and anonymous questionnaire. We used the validated versions of the brief HIV knowledge questionnaire and the Health Care Provider HIV/AIDS Stigma Scale.

Results: A total of 216 medical students completely answered the questionnaire. Overall, most participants demonstrated a good level of HIV-related knowledge (88.4%). Good HIV knowledge, higher parental education and involvement in community or associative activities were significantly associated with lower stigma levels. Interestingly, using multivariate analyses, poor parental educational level and being stigmatized were independently associated with higher prejudice scores. This student-centered learning approach was revealed to be effective in significantly improving HIV-related knowledge and reducing HIV stigma among medical students.

Conclusion: Our findings emphasize the value of integrating active learning methods into the medical curriculum to prepare future healthcare providers to deliver stigma-free care in order to promote ethical and inclusive behavior toward PLHIV.

Keywords: Stigma; HIV; Medical student; Education; Tunisia.

Introduction

The Human Immunodeficiency Virus (HIV) continues to be a significant global public health challenge. In 2023, it was reported that approximately 630,000 people died from HIV-related illness worldwide (1). This disease has a significant economic effect which includes direct medical costs, lost productivity, and public health expenditures. In fact, according to the joint United Nations Program on HIV/AIDS (UNAIDS), the annual global cost of HIV/AIDS is estimated to be over $30 billion (2) . Despite major advancements in treatment and prevention, HIV continues to be a major health challenge especially in low and middle-income countries (LMIC). A study published in 2023 revealed an increasing rate in HIV incidence, mortality, and disability-adjusted life years across the Middle East and North Africa (MENA) region (3). In Tunisia, the adult HIV prevalence is estimated at 0.1%, with approximately 8,000 people living with the virus in 2022 (4).

Several factors are associated with challenges in HIV care, including limited health education, socioeconomic disparities, inadequate health literacy and persistent knowledge gap (5,6). Stigma and discrimination against people living with HIV (PLHIV) particularly from healthcare providers continues to be a significant barrier discouraging individuals from seeking necessary care (7,8). According to a qualitative study conducted in Tunisia (9), healthcare provider misconceptions about this disease are one of the major leading causes to stigmatization and thus hinder combatting the HIV epidemic. In fact, HIV discrimination manifests as reluctance to provide care to PLHIV and use of extra precautionary measures resulting in delayed diagnosis and poor treatment adherence(10). Sexual education remains a taboo and HIV infection is wrongly seen as a divine punishment and a consequence of immoral behavior especially in MENA countries (11). This knowledge gap fuels stigma both among the general population and healthcare professionals (12,13). However, HIV stigma is still underexplored in developing countries compared to developed ones, particularly among healthcare workers. For instance, a 2021 study in Sana'a, Yemen revealed discriminatory attitudes among healthcare workers stemming from a lack of institutional policies against HIV stigma (14,15).

Hence, educating healthcare providers is crucial in assessing their knowledge gap and reducing stigmatizing practices. Several studies have shown that the impact of training healthcare professionals on HIV transmission, prevention and treatment can improve their attitudes and reduce stigma(16). According to a recent study conducted among healthcare workers in Vietnam (17), focused education programs raised engagement in HIV care and decreased

stigma. It highlights the importance of educational trainings in improving healthcare worker knowledge and attitudes toward PLHIV. Addressing this issue among medical students in the medical curriculum is fundamental for establishing a generation of future healthcare professionals who can provide equitable and compassionate care to PLHIV. Indeed, medical student attitudes and knowledge about HIV are shaped during their training, making this population a key target for interventions aimed at reducing stigma(18). However, most research on HIV-related stigma was among practicing clinicians, with limited focus on medical students, particularly in LMICs. A recent study in Saudi Arabia assessed this gap, reporting misconceptions and poor HIV knowledge among medical students. Thus, it is important to establish educational interventions among pre-clinical medical students.

Furthermore, while educational interventions have shown significant effects in reducing stigma, the effectiveness of active learning methods remains underexplored in the context of HIV (19). For instance, a thematic review revealed that the student-centered learning (SCL) method has the ability to foster deeper engagement, empathy and understanding by placing learners at the center of the educational process preparing them for professional challenges (20).

Given these data, the current study aimed to assess the impact of active training (workshops) on reducing stigma against PLHIV among fifth medical students in the Faculty of Medicine of Monastir (Tunisia) and to explore its related factors.

Method

Sampling and data collection:

We performed a quasi-experimental study during the academic years 2023–2024 and 2024–2025 among all fifth-year students in the Faculty of Medicine of Monastir (Tunisia), who have one remaining year of clinical training at the hospital during which they are responsible for patient care. The recruitment period for the first academic year was from 15/09/2023 to 31/05/2024, and for the second academic year from 15/09/2024 to 31/05/2025. The total duration of teaching during the academic year was 10 months.

A total of 205 students participated in our study, following the precedent set by a similar study by Al-Fadhli et al. (21). Their survey assessed the impact of HIV stigma-reduction workshops on healthcare providers in two tertiary-care hospitals and three healthcare colleges in central India, including 650 participants, of whom 205 were medical students (21). Despite different contexts, the intervention and population are similar, making it a relevant sample size reference.

Every week, a 2-hour workshop about HIV knowledge and practices was conducted for groups of 10 medical students at the Department of Preventive and Community Medicine within the faculty of Medicine of Monastir. The teacher, a professor of medicine, facilitated the sessions based on presentations and interactive lectures.

The intervention consisted of animating these workshops based on the SCL approach (20). This method shifts the traditional focus from instructor-led teaching to one that places students at the heart of the learning experience. It focuses on courses and classroom-based role-plays lectures, and open discussions prepared and delivered actively by students.

We evaluated HIV knowledge and practices as well as stigma attitudes towards PLHIV before and after each workshop using self-administered and validated measurement tools.

Measurement tools:

The study instrument in the current study was a self-administered questionnaire, and consisted of three sections as follows:

- **Knowledge about HIV among medical students**

To assess HIV knowledge, we used the validated French version of the **brief HIV knowledge questionnaire** (HIV-KQ-18). This questionnaire was originally developed by Carey et al. (2004) and was later validated in French (22). This tool is self-administered, which is designed

to assess general knowledge about HIV among medical students, modes of transmission, prevention methods and common misconceptions of the disease. It includes 18 yes or no statements evaluating attitudes and practices. Only five of the items are true (items 1, 4, 11, 14 and 17). The correct response is coded one, whereas the incorrect answers receive zero points with a maximum score obtainable for knowledge being 18 marks. Having a score of 14 or higher indicates a good HIV knowledge level, whereas a score below 14 signifies poor knowledge (23).

Stigma attitudes towards PLHIV among medical students

To evaluate stigma attitudes towards PLHIV, we used the validated instrument "**Health Care Provider HIV/AIDS Stigma Scale** (HPASS)" (24). The HPASS consists of a 30-item structured questionnaire. Each item is an example of a scenario that physicians might encounter in their future professional life or certain beliefs regarding PLHIV. In each statement, responders choose from six possible answers ‘’ strongly disagree, ‘’ ‘’slightly disagree ‘’ disagree” ‘’agree”, ‘’slightly agree” or ‘’ strongly agree”. These responses are assigned corresponding scores of 0, 1, 2, 3, 4, 5, respectively. This tool evaluates three dimensions (25): prejudice, stereotyping and discrimination, divided as follows:

- **Prejudice** (Items 1 to 13): These items reflect emotional reactions and negative attitudes towards PLHIV, including feelings of fear, blame or discomfort.
  A high score in this domain represents a stronger prejudicial attitude.
- **Stereotyping** (Items 14 to 23): These items evaluate negative beliefs towards PLHIV; assumptions about their behavior, lifestyle, or character. Stereotyping reflects cognitive biases often rooted in misinformation or cultural stigma
- **Discrimination** (Items 24 to 30): These items refer to discriminatory behavior or intentions, such as avoidance or unequal treatment of PLHIV. High scores indicate a greater likelihood of engaging in discriminatory actions.

In this section, we did not use predefined cut-off values to categorize participants into "low" or "high" stigma groups. Instead, the total score presented by the cumulative level of stigma, was considered as a continuous measure, with higher scores reflecting greater levels of stigma whether in the form of prejudice, stereotyping, or discriminatory behaviors. Comparing the mean scores before and after the intervention allows us to identify patterns or shifts in stigma and thus evaluate the impact of the active learning method (26).

- **Sociodemographic characteristics and sexual practices of participants.**

We collected data about sociodemographic characteristics and sexual practices to assess the potential influence of these factors on HIV related stigma. These questions were added based on a literature review about factors associated with HIV stigma. In the sociodemographic characteristics section, the collected data comprises: age, gender and parental educational level. Parental educational level was recorded into two categories: High educational level (at least one parent has completed higher education) and poor educational level (both parents have education levels below high school). We also asked about involvement in associative activities, HIV unit or screening campaigns and having received HIV-related training. The source of information on sexual and reproductive practices was grouped into reliable sources (Parents, Siblings, Cousins, School/University) and unreliable sources (Friends, Media, Internet). Data about experiencing stigma and sexual practices including: Previous sexual experience, condom protection, substance use during sex and sexually transmitted infections (STI) screening were collected.

Cultural context: Tunisia is a Muslim-majority country, and topics such as sexual behavior and HIV are often socially sensitive or somewhat taboo. In this context, HIV is generally associated with high-risk sexual practices, which may influence participants' willingness to report certain behaviors or attitudes honestly. This consideration was taken into account when designing the questionnaire and interpreting the results.

Statistics analysis:

Data entry and analysis were conducted using the Statistical Package for social science (SPSS); Version 23.0. Qualitative variables were represented by effectives and percentages. After normal distribution testing, continuous variables were expressed as the mean plus or minus the standard deviation (mean ± SD).

To compare variables with HIV knowledge levels, we used the Chi 2 test ($\chi 2$) or Fisher exact test since we compared percentages. To determine factors associated with stigma score and its three dimensions, we performed a univariate analysis using the student test to compare two independent means. The multivariate analysis via the linear regression model was conducted to identify independent factors associated with stigma scores.

Regarding the evaluation of the impact of the active workshop, dependent sample t-tests were performed to compare the mean scores of HIV knowledge and stigma before and after the active training method at the significant level of 0.05. Also, we calculated the Relative Reduction

(Percentage Change): as the difference between post-intervention and pre-intervention means, divided by the pre-intervention mean, and multiplied by 100.

Ethical considerations:

Participation in our study was voluntary. All participants provided informed consent and the study was anonymous. Students responded to the questionnaire in separate and distant tables. The data obtained from the students was solely used for the study purpose and was not included in the final score of the exam. The authors had no access to information that could identify students after data collection. The Ethic Committee of the University Hospital in Mahdia (CEM 2024-02-08) approved the study protocol.

Results

Sociodemographic characteristics and sexual practices of participants:

A total of 216 medical students answered the questionnaire completely with a response rate of 43.2%. The mean age of participants was 23 years±0.84 with a majority of female (63%). Participant background demographics and practices are outlined in Table 1.

Table 1: Sociodemographic characteristics and sexual practices among medical students.

Variables	Categories	Effective	Percentage
**Educational level of parents (N=216)**	High	157	72.7%
	Poor	59	27.3%
**Involvement in associative activities, HIV unit or screening campaigns (N=216)**	Yes	50	23.1%
	No	166	76.9%
**Source of sexual knowledge (N=216)**	Reliable	81	37.5%
	Unreliable	135	62.5%
**Experiencing stigma or discrimination (N=216)**	Yes	31	14.4%
	No	185	85.6%
**Previous sexual experience (N=216)**	Yes	28	13%
	No	188	87%
**Condom protection (N=28)**	Yes	16	57.1%
	No	12	42.9%
**Substance use during sex (N=28)**	Yes	24	50%
	No	24	50%
**STI screening (N=216)**	Yes	5	2.3%
	No	211	97.7%

Pre-intervention HIV stigma among medical students and its related factors:

The majority of participants presented good knowledge levels (88.4%). Table 2 presents the results of univariate analysis on variables influencing the total stigma score among medical students. In fact, individuals having good HIV knowledge, high parental educational level and those who were involved in associative activities, HIV units or screening campaigns exhibited lower stigma scores (Table 2).

Table 2: Factors influencing the total stigma score among medical students: Univariate analysis

Variables	Categories	Mean stigma score±SD	P value
**HIV knowledge (N=216)**	Good	**59.34±22.2**	**0.01**
	Poor	**69.88±17.63**	
**Gender (N=216)**	Male	59.76±24.65	0.685
	Female	61.02±20.31	
**Educational level of parents (N=216)**	High	**58.65±22.36**	**0.037**
	Poor	**65.64±20.12**	
**Involvement in associative life HIV unit or screening campaigns (N=216)**	Yes	**53.14±22.07**	**0.006**
	No	**62.79±21.47**	
**Source of sexual knowledge (N=216)**	Reliable	60.26±23.67	0.876
	Unreliable	60.74±20.93	
**Experiencing stigma or discrimination (N=216)**	Yes	64.48±24.11	0.283
	No	60±21.56	
**Previous Sexual experience (N=216)**	Yes	61.43±21.04	0.213
	No	54.67±26.96	
**Condom protection (N=28)**	Yes	46.56±25.96	0.065

	No	65.5±25.33	
**Substance use during sex (N=28)**	Yes	55.54±28.17	0.576
	No	59.46±19.2	
**STI screening (N=216)**	Yes	50.4±20.41	0.296
	No	60.8±22	

Prejudice related factors

Table 3 highlights variables influencing prejudice among dimension among medical students through univariate analysis. Having a high educational level of parents and being involved in associative activities, HIV units or screening campaigns were associated with lower prejudice scores (Table 3).

Table 3: Factors influencing prejudice dimension among medical students: Univariate analysis

Variables	Categories	Mean prejudice score±SD	P value
**HIV knowledge (N=216)**	Good	26.68±11.47	0.094
	Poor	30.72±9.78	
**Gender (N=216)**	Male	26.44±12.684	0.489
	Female	27.55±10.51	
**Educational level of parents (N=216)**	High	**25.97±11.25**	**0.013**
	Poor	**30.27±11.07**	
**Involvement in associative activities, HIV unit or screening campaigns (N=216)**	Yes	**22.7±11.23**	**0.001**
	No	**28.49±11.06**	
**Source of sexual knowledge(N=216)**	Reliable	26.65±12.43	0.621
	Unreliable	27.44±10.67	

**Experiencing stigma or discrimination (N=216)**	yes	**30.93±13.74**	**0.044**
	No	**26.51±10.8**	
**Previous Sexual experience (N=216)**	Yes	25.28±13.66	0.353
	No	27.42±10.97	
**Condom protection (N=28)**	Yes	21.93±11.9	0.137
	No	29.75±15.06	
**Substance use during sex (N=28)**	Yes	26.04±13.86	0.972
	No	26.16±10.15	
**STI screening (N=216)**	Yes	25.2±10.1	0.699
	No	27.19±11.38	

Multivariate linear regression showed that lower parental educational level and experiences of stigma or discrimination were independently associated with higher HIV prejudice scores (for more details, see table4).

Table 4: Factors influencing prejudice among medical students: Multivariate linear regression analysis

Related Factors to HIV prejudice	Standard Errors	P value	B coefficient	IC95%
**Educational level of parents (N=216)**	5.033	0.045	0.359	0.27 - 21.05
**Experiencing stigma or discrimination (N=216)**	5.925	0.042	0.364	0.53 - 24.98

Stereotype related factors:

Table 5 shows variables influencing stereotype among medical students through univariate analysis.

Table 5: Factors influencing stereotype among medical students: Univariate analysis

Variables	Categories	Mean stereotype score±SD	P value

**HIV knowledge (N=216)**	Good	21.79±8.15	0.166
	Poor	24.2±8	
**Educational level of parents (N=216)**	High	**21.34±8.2**	**0.033**
	Poor	**24±7.8**	
**Involvement in associative life HIV unit or screening campaigns (N=216)**	Yes	22.54±8.01	0.121
	No	20.5±8.52	
**Previous Sexual experience (N=216)**	Yes	19.67±10.1	0.097
	No	22.42±7.8	
**Condom protection (N=28)**	Yes	**16.31±10.34**	**0.039**
	No	**24.16±8.13**	

Discrimination dimension:

No variables influencing the discrimination dimension among medical students were found through univariate analysis in our sample.

Impact of SCL method on HIV knowledge and stigma against PLHIV:

Table 6 shows the score trends before and after the active workshops (knowledge, stigma, prejudice, stereotype and discrimination scores).

Regarding HIV knowledge, the overall score increased significantly from 15.57±1.61 to 16.3±1.5. A notable reduction was observed in the stigma score following the SCL method. In fact, the mean score significantly decreased from 60.56±21.94 to 52.64±22. Similar results were found for all stigma dimensions (Table 6).

Table 6: Impact of SCL method on HIV knowledge and stigma dimensions (N=216)

Variable	Mean knowledge and stigma scores ±SD		P value	Relative change (%)
	Before	After		
**Knowledge score**	**15.57±1.61**	**16.3±1.5**	**<0.001**	**+4.69**
**Total Stigma score**	**60.56±21.94**	**52.64±22**	**<0.001**	**-13.08**
**Prejudice score**	**27.14±11.34**	**22.57±11.09**	**<0.001**	**-16.84**
**Stereotype score**	**22.07±8.16**	**20.45±8.51**	**<0.001**	**-7.34**
**Discrimination score**	**11.34±7.54**	**9.61±7.29**	**<0.001**	**-15.26**

Discussion:

To our knowledge, our study is among the first in North Africa to focus on stigma among medical students against PLHIV and to assess the impact of the SCL method on both knowledge and stigma. In fact, we found that of all stigma dimensions, prejudice showed significant associations in multivariate analysis. Interestingly, poor parental educational level and being stigmatized were independently associated with higher prejudice scores. In addition, we found that the SCL method was effective in significantly improving HIV-related knowledge as well as in reducing stigma scores among medical students.

We found that the majority of participants were not involved in HIV related activities or screening campaigns (76.9%). This result is in contrast to findings in Hong Kong showing that 72.1% of final-year medical students were exposed to HIV patients during attachment to an HIV clinic in the late 2000s, compared with 39.3% in the mid-2010s (27). The significantly lower exposure in Tunisia (23.1%) compared to Hong Kong likely stems from structural and institutional differences. Tunisian HIV care remains highly centralized in a few specialized units. Furthermore, pervasive socio-cultural stigma in Tunisia often necessitates a higher degree of patient anonymity, which, combined with strict confidentiality protocols, limits the integration of HIV-positive patients into general undergraduate clinical training. More broadly, in MENA countries, stigma, cultural taboos and lack of structured programs may contribute to the low involvement rates (11). In our study, most medical students reported having an unreliable source of sexual knowledge (85.5%). However, studies in Iran and Greece reported greater use of reliable source (28). This may be influenced by cultural and societal differences. In fact, in Tunisia, sexual health topics are still taboos leading younger individuals to seek false information elsewhere.

Among students who were sexually active in our sample, only 57.1% use condoms for protection. This contrasts with results among Mexican medical students in 2024, where a higher proportion of students reported condom use during vaginal intercourse (83.6 % in men and 67.7% in women) (29). However, condom use cannot be attributed only to the level of sexual health education. Indeed, other influencing factors such as partner preference, access to condoms, personal and cultural attitudes affect the use of this protective measure (30). We found that only 2.3% of participants underwent STI screening. Similarly, in a 2023 study conducted in Warsaw, Poland, Only 21.43% of participants had ever been tested for HIV (31). Several explanations could be cited such as low sexual encounters, fear of being stigmatized for testing and lack of HIV related knowledge (31). In our cultural context, sexual health remains a

sensitive topic, which may explain the very low STI screening rates and reliance on unreliable sources of information. These findings highlight the urgent need for broader, culturally appropriate sexual health education among medical students.

As for HIV knowledge, findings in our study showed a good knowledge level among 88.4% of medical students. In fact, in the Tunisian medical curriculum, students start learning about HIV and its impact early in their education. This is in accordance with results reported in Iran and in countries from both sub-Saharan Africa and North Africa (32,33). Nevertheless, it remains essential to sustain efforts aimed at further enhancing HIV knowledge by screening the related factors and by developing targeted interventions in educational medical trainings. To the best of our knowledge, our study is the first to focus on stigmatizing attitudes towards PLHIV among medical students in Tunisia. As medical students play a key role in future care delivery, it is crucial to assess stigma and its related factors. Students with higher scores on HIV-related knowledge exhibit lower stigma rates. These results align with studies conducted in Yemen and Indonesia among healthcare professionals, where poor knowledge of HIV was linked to higher rates of stigma (34,35). However, an Indonesian study suggested that physicians exhibited both the highest HIV knowledge and the highest levels of stigma (36). Thus, combatting stigma requires interventions that transcend knowledge and highlight cultural beliefs and other factors influencing healthcare professionals' attitudes. We found that female participants exhibit a higher stigma score compared to males. These results align with findings from an Iranian study (37) and contradicts with another study from the USA (38). However, the gender difference observed in our study was not statistically significant and should therefore be interpreted with caution. In certain sociocultural contexts where discussions surrounding sexuality and HIV may be more socially constrained for women, gender-related norms could potentially influence attitudes. This hypothesis warrants further investigation in larger and more diverse samples. In our survey, students who were involved in associative activities, HIV units and screening campaigns exhibited a lower stigma score. Likewise, a study conducted in Jordan among medical students stated that pre-clinical students had a significantly more negative attitude towards PLHIV compared to their clinical counterparts (39). Likewise, from a study carried out among students from Hong Kong, it was found that pupils who were not willing to provide care for PLHIV had less clinical experience with HIV patients (27). We found that individuals untested for HIV tend to have a higher stigma score. These findings align with results from Indonesia (35). One possible explanation is that during HIV testing and counseling, individuals might gain great HIV knowledge and undergo the emotional experience of diagnosis. Our study

found no significant association between the reliability of the source and stigma rates. However, although the effect is weak, the media was found to be an enhancing factor for HIV knowledge and a reducing factor of HIV stigma in Iran (40). The media is a powerful tool for combatting stigma against PLHIV when it is guided by accurate information about the disease. However, in many conservative societies, PLHIV continue to face negative representations in the media, reinforcing panic and misconceptions. Thus, it is urgent to implement media-based awareness and evidence-based education(41).

HIV prejudice refers to negative feelings, judgments, and attitudes towards PLHIV rooted in negative beliefs and often reinforced by fear of the disease (25). We found that students with better HIV-related knowledge showed lower levels of prejudice. Similarly, dental healthcare providers in Pakistan with limited HIV-related knowledge tended to hold more negative attitudes towards HIV (42). Participation in HIV-related workshops or screening campaigns was linked to a lower prejudice score in our sample. Likewise, trainings on HIV stigma and/or discrimination was associated with less negative attitudes in healthcare providers in Germany (43). This highlights the importance of structured educational interventions for reducing prejudice among healthcare professionals. Beyond the univariate analysis, multivariate analysis of stigma dimensions revealed specific predictors of prejudice. Notably, previous personal experience of being stigmatized and poor parental educational level were independently associated with higher prejudice scores. Limited research has explored how an individual's own history of being stigmatized shapes their attitudes toward PLHIV. However, based on results reported in Zambia, healthcare professionals who did not stigmatize but witnessed stigmatizing attitudes among their coworkers, were more likely to hold critical and stigmatizing attitudes (44). We did not find evidence of an association between the parental educational level of students and their attitude towards PLHIV. On the other hand, in Indonesia, belonging to the richest or rich economic group was associated with higher levels of stigma against PLHIV (45). In contrast, a Ugandan study reported that wealthier individuals were less likely to endorse social distance and stigma against PLHIV(46). These findings suggest that neither wealth nor parental education level predicts young people's attitudes. To the best of our knowledge, this is the first study to report associated factors with prejudice scores. Nonetheless, factors such as relationship dynamics, personal convictions, and social barriers must also be taken into account when evaluating attitudes toward HIV. Assessing related factors to HIV stigma into medical curriculum is needed to prepare future professionals into delivering compassionate and equitable care to PLHIV and to combat HIV decease (47).

Reducing stigma is essential to improving healthcare outcomes and care delivery especially among healthcare professionals (17,48). Through the years, Depersonalization of patient cases and lack of diverse learning methods have limited student engagement and empathy. Traditional learning approaches often fail to nurture humanistic and relational skills. As a result, various learning methods have been used in different curricula and medical education such as problem-based learning, Case-based discussions, and flipped-classroom methods. Another innovative approaches involve Game-based learning, Team-based learning , Simulation-based learning, and peer teaching. They all embody the concept of SCL placing the learner at the core of the educational process (49). It involves active participation where students engage in role-playing scenarios, open discussions and lectures. Students take on the roles of actors in various role-playing scenarios and are often placed in patient and doctor shoes (50). Further, a range of educational interventions has been employed for HIV-stigma reduction (16). Thus, we aimed to assess the impact and effectiveness of this method in enhancing HIV-related knowledge and reducing stigma among medical students. In our study, after the SCL approach, the overall HIV knowledge score significantly improved. A significant decline in HIV stigma scores was also noted and similar improvements were observed across all stigma dimensions. For instance, similar Tunisian data showed that both active participants and observers achieved significant gains in knowledge scores after a role-play simulation (51). Similarly, a Tunisian study assessed the impact of the SCL among medical students in the faculty of medicine of Monastir. Significant improvement in hand hygiene and biomedical waste management after the SCL intervention (52).

Beyond its implication in general medical subjects, the SCL method has also been explored in our primary focus, enhancing HIV-related knowledge and reducing stigma against PLHIV. Given the HPASS scoring range, the observed 7.9-point decrease reflects a tangible reduction in stigmatizing attitudes, indicating that the intervention may have practical educational relevance in shaping future healthcare professionals' perspectives toward PLHIV. For instance, a peer-led education intervention among secondary school students in Sudan showed a significant improvement in participant knowledge, attitudes and practices regarding HIV. This study highlights the effectiveness of SCL and peer-based approaches in promoting HIV awareness and reducing HIV stigma among medical students (53). Similarly, a pilot study in the United States assessed the Facilitated Resilience and Equity through Stigma-reduction and Health workshop, a reduction of HIV-related stigma intervention among healthcare workers. During the workshop, participants interacted through discussions and role-playing. Findings

showed a significant reduction in stigma and a significant HIV-related knowledge increase (54). In addition, in China, a web-based game was developed to reduce HIV-related stigma. The game used participatory gamification and storytelling to immerse university students in the lives of PLHIV. This approach enhanced empathy-building, active learning through problem solving and reflection (55). In addition, our study results align with a previous Indian study conducted among healthcare professionals (56). The HIV-related stigma reduction workshops in Central India significantly impacted the knowledge and attitudes of healthcare providers and students. These results were assessed through a pre and post-test similar to our intervention.

To our knowledge, our study is the first globally and in the MENA region to simultaneously integrate the HIV-KQ-18 and the HPASS in a single research framework among medical students. HIV knowledge and stigma are critical issues in the MENA region, where persistent misconceptions, cultural taboos, and deeply rooted stigma continue to hinder efforts in HIV prevention and patient care. Addressing these challenges among medical students as future healthcare providers is an essential urgent step toward a long-term stigma-free environment and improved quality of life for PLHIV. It is essential to form medical graduates with a solid foundation in professional ethics, empathy, and a clear understanding of patient rights and confidentiality, ensuring they are prepared to deliver respectful and stigma-free care for patients. Uniquely, our study also incorporates a before-and-after intervention design, embedding active training and SCL modules within the medical education curriculum.

These results should be considered in light of the study limitations. First, it was a single-center study with a limited sample size. Since the study was conducted among medical students from one institute, findings cannot be generalized to broader populations of medical or healthcare students. Similar constraints were observed in a Vietnamese study among medical students who assessed HIV knowledge and stigma among a limited group of 200 medical students (57). In addition, the Faculty of Medicine of Monastir operates within a specific educational, institutional, and sociocultural context that may influence students' attitudes toward PLHIV. Tunisia is part of the MENA region, where HIV-related stigma is often shaped by religious norms, cultural perceptions surrounding sexuality, and societal attitudes toward key populations. These contextual factors may influence baseline stigma levels as well as responsiveness to educational interventions. Furthermore, variations in curriculum content, clinical exposure to PLHIV, and institutional culture across medical faculties may affect the transferability of our findings to other settings, both nationally and internationally. To enhance the generalizability and reliability of the findings, it is recommended to generalize our

intervention to all healthcare institutes on a national scale for reliable results. Secondly, one important limitation of this study was the lack of future follow-up of the impact of the intervention. While the training aimed to reduce HIV stigmatizing attitudes among medical students, changes in practices among healthcare professionals were not evaluated. The current research did not conduct long-term follow-up surveys to verify the sustainability of the training, making it impossible to determine whether the training truly had a positive impact on the participant behavior against PLHIV. This gap between theoretical training and healthcare practices is highlighted in an Indian study, which emphasized the absence of stage-based follow-up to assess the effectiveness of the reduction workshops (56). Future studies should integrate multiple follow-up stages for a deeper understanding of how knowledge and attitudes evolve and potentially translate into lasting behavioral change. Furthermore, because stigma was measured immediately after the intervention, we cannot determine whether the observed reduction reflects a sustained change in attitudes or a short-term response. Future research is planned to include longitudinal follow-up assessments at multiple time points to evaluate the durability of attitude change over time. Thirdly, in our study, the relatively low response rate (43.2%) may reflect potential bias, as some students may have chosen not to participate due to the sensitive nature of certain questions related to sexuality and religious or cultural norms.

Conclusion

Our findings underscore the urgent need to integrate continuous anti-stigma education into medical curricula through active learning approaches. Interventions must be introduced early in the medical curriculum and backed by strong institutional commitment. Moreover, such efforts must be sustained and extend beyond healthcare settings to target the general population fostering an informed and nondiscriminatory society. Within the healthcare environment and the general population, a graduated accountability system should be introduced to address stigma early on.

Acknowledgements:

We would like to thank all the participants of the study.

Data Availability Statement:

All data underlying the findings described in this manuscript are fully available within the Supporting Information file. The Excel dataset (S1_Data.csv) can be freely accessed.

References

1. World Health Organisation. HIV [Internet]. 2025 [cited 2025 Feb 3]. Available from: https://www.who.int/data/gho/data/themes/hiv-aids?utm_source=chatgpt.com

2. UNAIDS. Global HIV & AIDS statistics — Fact sheet [Internet]. 2025 [cited 2025 Feb 3]. Available from: https://www.unaids.org/en/resources/fact-sheet

3. Khorrami Z, Balooch Hasankhani M, Khezri M, Jafari-Khounigh A, Jahani Y, Sharifi H. Trends and projection of incidence, mortality, and disability-adjusted life years of HIV in the Middle East and North Africa (1990–2030). Sci Rep. 2023 Aug 24;13(1):13859.

4. UNAIDS. Tunisia [Internet]. 2025 [cited 2025 Feb 5]. Available from: https://www.unaids.org/en/regionscountries/countries/tunisia

5. Kumah E, Boakye DS, Boateng R, Agyei E. Advancing the Global Fight Against HIV/Aids: Strategies, Barriers, and the Road to Eradication. Ann Glob Health [Internet]. [cited 2025 May 13];89(1):83.

6. Bouabida K, Chaves BG, Anane E. Challenges and barriers to HIV care engagement and care cascade: viewpoint. Front Reprod Health [Internet]. 2023 Jul 20 [cited 2025 May 13];5.

7. European Centre for Disease Prevention and Control. HIV stigma in healthcare settings: need for increased knowledge among healthcare workers and improved facility-level guidelines. Stockholm: ECDC; 2024 [cited 2025 May 13]. Available from: https://www.ecdc.europa.eu/en/news-events/hiv-stigma-healthcare-settings-need-increased-knowledge-among-healthcare-workers-and

8. Crockett KB, Turan B, Whitfield S, Kay ES, Budhwani H, Fifolt M, et al. Patient and Provider Perspectives on HIV Stigma in Healthcare Settings in Underserved Areas of the US South: A Mixed Methods Study. AIDS Behav [Internet]. 2022 Jan [cited 2025 May 13];26(Suppl 1):112–24.

9. Zribi M, Mansour NB, Moussa H, Hassine HB, Aounallah-Skhiri H. Experiences and perceptions of health professionals towards the quality of care for people living with HIV in Tunisia: a qualitative study. Pan Afr Med J [Internet]. 2023 Sep 6 [cited 2025 Feb 5];46:4.

10. Armoon B, Fleury MJ, Bayat AH, Fakhri Y, Higgs P, Moghaddam LF, et al. HIV related stigma associated with social support, alcohol use disorders, depression, anxiety, and suicidal ideation among people living with HIV: a systematic review and meta-analysis. Int J Ment Health Syst [Internet]. 2022 Mar 4 [cited 2025 Feb 3];16(1):17.

11. Kteily-Hawa R, Hawa AC, Gogolishvili D, Al Akel M, Andruszkiewicz N, Vijayanathan H, et al. Understanding the epidemiological HIV risk factors and underlying risk context for youth residing in or originating from the Middle East and North Africa (MENA) region: A scoping review of the literature. PLoS ONE [Internet]. 2022 Jan 7 [cited 2025 Apr 13];17(1):e0260935.

12. Aldhaleei WA, Bhagavathula AS. HIV/AIDS-knowledge and attitudes in the Arabian Peninsula: A systematic review and meta-analysis. J Infect Public Health [Internet]. 2020 Jul 1 [cited 2025 May 13];13(7):939–48.

13. Elghazaly A, AlSaeed N, Islam S, Alsharif I, Alharbi L, Al Ashagr T, et al. Assessing the knowledge and attitude towards HIV/AIDS among the general population and health care professionals in MENA region. PLOS ONE [Internet]. 2023 Jul 27 [cited 2025 May 13];18(7):e0288838.

14. Attal BA, Al-Rowaishan KM, Akeel AA, AlAmmar FK. HIV stigma in the teaching hospitals in Sana'a, Yemen: a conflict and low-resource setting. BMC Public Health [Internet]. 2021 Oct 5 [cited 2025 Apr 18];21:1793.

15. Rizk NA, Helou RE, AlMukdad S, Fahme S, Mumtaz GR, Chemaitelly H, et al. Has UNAIDS left the Middle East and north Africa behind? Lancet HIV [Internet]. 2023 Oct 1 [cited 2025 May 13];10(10):e630–2.

16. Ferguson L, Gruskin S, Bolshakova M, Rozelle M, Yagyu S, Kasoka K, et al. Systematic review and quantitative and qualitative comparative analysis of interventions to address HIV-related stigma and discrimination. AIDS Lond Engl [Internet]. 2023 Nov 1 [cited 2025 May 1];37(13):1919–39.

17. Pollack TM, Duong HT, Nhat Vinh DT, Phuong DT, Thuy DH, Nhung VTT, et al. A pretest-posttest design to assess the effectiveness of an intervention to reduce HIV-related stigma and discrimination in healthcare settings in Vietnam. J Int AIDS Soc. 2022 Jul;25 Suppl 1(Suppl 1):e25932.

18. Alshouibi E, Alaqil F. HIV-Related Discrimination among Senior Dental Students in Jeddah. J Int Soc Prev Community Dent [Internet]. 2019 [cited 2025 May 13];9(3):219–24.

19. Taggart T, Taboada A, Stein JA, Milburn NG, Gere D, Lightfoot AF. AMP!: A Cross-site Analysis of the Effects of a Theater-based Intervention on Adolescent Awareness, Attitudes, and Knowledge about HIV. Prev Sci Off J Soc Prev Res [Internet]. 2016 Jul [cited 2025 May 13];17(5):544–53.

20. (PDF) A thematic review on student-centred learning in the studio education [Internet]. [cited 2025 Feb 6]. Available from: https://www.researchgate.net/publication/339640951_A_thematic_review_on_student-centred_learning_in_the_studio_education?utm_source=chatgpt.com

21. Machowska A, Bamboria BL, Bercan C, Sharma M. Impact of 'HIV-related stigma-reduction workshops' on knowledge and attitude of healthcare providers and students in Central India: a pre-test and post-test intervention study. BMJ Open [Internet]. 2020 Apr 1 [cited 2025 Jan 24];10(4):e033612.

22. Carey MP, Schroder KEE. Development and Psychometric Evaluation of the Brief HIV Knowledge Questionnaire. AIDS Educ Prev Off Publ Int Soc AIDS Educ [Internet]. 2002 Apr [cited 2025 Feb 7];14(2):172–82.

23. Wagenaar BH, Sullivan PS, Stephenson R. HIV Knowledge and Associated Factors among Internet-Using Men Who Have Sex with Men (MSM) in South Africa and the United States. PLoS ONE [Internet]. 2012 Mar 12 [cited 2025 Jan 25];7(3):e32915.

24. Wagner AC, Hart TA, McShane KE, Margolese S, Girard TA. Health Care Provider Attitudes and Beliefs About People Living with HIV: Initial Validation of the Health Care Provider HIV/AIDS Stigma Scale (HPASS). AIDS Behav [Internet]. 2014 Dec 1 [cited 2025 Jan 24];18(12):2397–408.

25. Wagner AC, Hart TA, McShane KE, Margolese S, Girard TA. Health Care Provider Attitudes and Beliefs About People Living with HIV: Initial Validation of the Health Care Provider HIV/AIDS Stigma Scale (HPASS). AIDS Behav [Internet]. 2014 Dec 1 [cited 2025 Jan 24];18(12):2397–408.

26. Heidarpoor P, Mousavikashi S. Effectiveness of peer assisted learning in improving HIV knowledge, attitude, and perception among community health workers. Res Dev Med Educ [Internet]. 2023 Aug 29 [cited 2025 Jan 25];12(1):6–6.

27. HKMJ [Internet]. 2022 [cited 2025 Apr 13]. Serial surveys of Hong Kong medical students regarding attitudes towards HIV/AIDS from 2007 to 2017.

28. Lagadinou M, Spiliopoulou K, Paraskevas T, Gkentzi D, Assimakopoulos S, Katsakiori P, et al. Knowledge and Attitudes of Medical and Nursing Students in a Greek University Regarding Sexually Transmitted Diseases. Int J Environ Res Public Health [Internet]. 2024 Feb 22 [cited 2025 Apr 12];21(3):251.

29. Garcia-Romo GS, Pozo-Molina G, Reyes-Reali J, Mendez-Catala CF, Garrido E, Mendez-Cruz AR, et al. Prevalence of sexually risky behaviors among Mexican medical students. PLOS ONE [Internet]. 2024 May 6 [cited 2025 Apr 12];19(5):e0302570.

30. Lalo R, Theodhosi G, Breshanaj A. Health beliefs and barriers related to HIV prevention and screening among students of the University of Vlora: a cross-sectional study. BMC Public Health [Internet]. 2020 Aug 27 [cited 2025 Apr 14];20:1302.

31. Kowalska J, Cholewik M, Bieńkowski C, Maciejczyk A, Bursa D, Skrzat-Klapaczyńska A. Knowledge and Awareness of Risk Factors for HIV Infection and about HIV Testing among Medical Students in Warsaw. Viruses [Internet]. 2024 Sep 15 [cited 2025 Apr 12];16(9):1470.

32. Pourjam R, Rahimi Khalifeh Kandi Z, Estebsari F, Karimi Yeganeh F, Safari M, Barati M, et al. An Analytical Comparison of Knowledge, Attitudes, and Practices Regarding HIV/AIDS Among Medical and Non-Medical Students in Iran. HIVAIDS Auckl NZ [Internet]. 2020 Apr 28 [cited 2025 Apr 12];12:165–73.

33. Terra M, Okereke PU, Wandera F, Edith K, Kitonga MS, Ally AM, et al. HIV related knowledge and practices among undergraduate students in Africa: A cross-sectional multinational study. J Med Surg Public Health [Internet]. 2024 Aug 1 [cited 2025 Apr 16];3:100126.

34. Attal BA, Al-Rowaishan KM, Akeel AA, AlAmmar FK. HIV stigma in the teaching hospitals in Sana'a, Yemen: a conflict and low-resource setting. BMC Public Health [Internet]. 2021 Oct 5 [cited 2025 Jul 27];21:1793.

35. Fauk NK, Ward PR, Hawke K, Mwanri L. HIV Stigma and Discrimination: Perspectives and Personal Experiences of Healthcare Providers in Yogyakarta and Belu, Indonesia. Front Med [Internet]. 2021 May 12 [cited 2025 Apr 18];8:625787.

36. Hidayat J, Chen MY, Maulina R, Nurbaya S. Factors Associated With HIV-Related Stigma Among Indonesian Healthcare Workers: A Cross-Sectional Online Survey. J Nurs Res [Internet]. 2023 Oct [cited 2025 Apr 20];31(5):e295.

37. Tavakoli F, Karamouzian M, Rafiei-Rad AA, Iranpour A, Farrokhnia M, Noroozi M, et al. HIV-Related Stigma Among Healthcare Providers in Different Healthcare Settings: A Cross-Sectional Study in Kerman, Iran. Int J Health Policy Manag [Internet]. 2019 Oct 21 [cited 2025 Apr 18];9(4):163–9.

38. Stringer KL, Turan B, McCormick L, Durojaiye M, Nyblade L, Kempf MC, et al. HIV-Related Stigma among Healthcare Providers in the Deep South. AIDS Behav [Internet]. 2016 Jan [cited 2025 Apr 18];20(1):115–25.

39. Sallam M, Alabbadi AM, Abdel-Razeq S, Battah K, Malkawi L, Al-Abbadi MA, et al. HIV Knowledge and Stigmatizing Attitude towards People Living with HIV/AIDS among Medical Students in Jordan. Int J Environ Res Public Health [Internet]. 2022 Jan 10 [cited 2025 Apr 15];19(2):745.

40. Aghaei A, Mohraz M, Shamshirband S. Effects of media, interpersonal communication and religious attitudes on HIV-related stigma in Tehran, Iran. Inform Med Unlocked [Internet]. 2020 Jan 1 [cited 2025 Apr 20];18:100291.

41. Iacoella F, Gassmann F, Tirivayi N. Impact of mobile phones on HIV public stigma: a cross-sectional and pseudo-panel analysis from Ghana. BMJ Open [Internet]. 2022 Nov 8 [cited 2025 May 2];12(11):e062594.

42. Rana BK, Sarfraz M, Reza TE, Emmanuel F. A Cross-Sectional Study to Assess HIV/AIDS-Related Stigma and Its Drivers Among Dental Healthcare Providers in Islamabad, Pakistan. Cureus [Internet]. [cited 2025 Apr 27];15(10):e46769.

43. Jordans CCE, Vliegenthart-Jongbloed KJ, van Bruggen AW, van Holten N, van Beek JEA, Vriesde M, et al. Unmasking Individual and Institutional HIV Stigma in Hospitals: Perspectives of Dutch Healthcare Providers. AIDS Behav [Internet]. 2024 [cited 2025 Apr 27];28(9):3184–95.

44. Krishnaratne S, Bond V, Stangl A, Pliakas T, Mathema H, Lilleston P, et al. Stigma and Judgment Toward People Living with HIV and Key Population Groups Among Three Cadres of Health Workers in South Africa and Zambia: Analysis of Data from the HPTN 071 (PopART) Trial. AIDS Patient Care STDs [Internet]. 2020 Jan 1 [cited 2025 May 3];34(1):38–50.

45. Suantari D. Misconceptions and stigma against people living with HIV/AIDS: a cross-sectional study from the 2017 Indonesia Demographic and Health Survey. Epidemiol Health [Internet]. 2021 Nov 6 [cited 2025 May 3];43:e2021094.

46. Tsai AC, Venkataramani AS. The causal effect of education on HIV stigma in Uganda: evidence from a natural experiment. Soc Sci Med 1982 [Internet]. 2015 Oct [cited 2025 May 3];142:37–46.

47. Ebrahimi H, Shoorideh FA, Sohrabi MR, Ebrahimi M, Hosseini M. An Analysis of Approaches to Reduction of HIV Stigma across the World through educational interventions: A Scoping Review. Investig Educ En Enfermeria [Internet]. 2024 Apr 27 [cited 2025 Apr 28];42(1):e06.

48. Srinivasan K, Heylen E, Raj T, Nyblade L, Devadass D, Pereira M, et al. Reduction in stigma drivers partially mediates the effect of a stigma reduction intervention among nursing students in India: The DriSti cluster randomized controlled trial. J Acquir Immune Defic Syndr 1999 [Internet]. 2021 Feb 1 [cited 2025 Apr 28];86(2):182–90.

49. Berg E, Lepp M. The meaning and application of student-centered learning in nursing education: An integrative review of the literature. Nurse Educ Pract [Internet]. 2023 May 1 [cited 2025 Apr 6];69:103622.

50. Yousaf A, Moin H, Majeed S, Shafi R, Mansoor S. The positive impact of introducing modified directed self-learning using pre–small group discussion worksheets as an active learning strategy in undergraduate medical education. Med Educ Online [Internet]. [cited 2025 Apr 29];28(1):2204547.

51. Hammami O, Zaouche K, Kallel M, Nouira M. Évaluation des capacités d'apprentissage après la méthode du jeu de rôle: Comparaison des résultats de l'activité active et de l'observation. Tunis Médicale [Internet]. 2024 Nov [cited 2025 Apr 29];102(11):866–70.

52. Mlouki I, Ayed SB, Chebbi F, Rezg N, Khouildi A, Sassi AH, et al. Hand hygiene and biomedical waste management among medical students: a quasi-experimental study evaluating two training methods. BMC Med Educ [Internet]. 2023 Sep 4 [cited 2025 Jul 27];23:634.

53. Ali MHM, Osman OB, Ibrahim MAEM, Ahmed WAM. The Effect of AIDS Peer Health Education on Knowledge, Attitudes, and Practices of Secondary School Students in Khartoum, Sudan. AIMS Public Health [Internet]. 2015 Oct 22 [cited 2025 Apr 27];2(4):718–26.

54. Batey DS, Whitfield S, Mulla M, Stringer KL, Durojaiye M, McCormick L, et al. Adaptation and Implementation of an Intervention to Reduce HIV-Related Stigma Among Healthcare Workers in the United States: Piloting of the FRESH Workshop. AIDS Patient Care STDs [Internet]. 2016 Nov 1 [cited 2025 Apr 5];30(11):519–27.

55. Doroudi F, Ashourizadeh H, Moosapour S, Ganjizadeh A, Ranjbar-Shourabi A, Parnia A, et al. Gamified Educational Learning Path on HIV/AIDS Stigma and Discrimination (REDXIR): Design, Development and Pilot Study. Med J Islam Repub Iran [Internet]. 2023 Dec 16 [cited 2025 May 1];37:136.

56. Machowska A, Bamboria BL, Bercan C, Sharma M. Impact of 'HIV-related stigma-reduction workshops' on knowledge and attitude of healthcare providers and students in Central India: a pre-test and post-test intervention study. BMJ Open [Internet]. 2020 Apr 1 [cited 2025 Jan 24];10(4):e033612.

57. Platten M, Pham HN, Nguyen HV. Knowledge of HIV and factors associated with attitudes towards HIV among final-year medical students at Hanoi medical university in Vietnam. BMC Public Health [Internet]. 2014 Mar 20 [cited 2025 May 10];14:265.

Supporting Information:

S1 Data. Anonymized Excel dataset (.scv). This file contains the data used to conduct the analyses presented in this study.

Questionnaire

Click here to access/download
Supporting Information
Questionnaire.docx

Supporting information

Click here to access/download
Supporting Information
S1_Data.csv

Stigma against People Living with Human Immunodeficiency Virus: A quasi-experimental evaluation of Active Stigma Reduction Workshops among Medical Students in Tunisia

Authors: Imen Mlouki[1,2,3], Emna Baoueb[1], Emna Hariz[2,3*], Aya Ajmi Blout[1,2], Haythem Ayachi[1], Ahmed Moustafa[4], Sana El Mhamdi[1,2,3].

1Preventive and Community Medicine, Faculty of Medicine of Monastir, University of Monastir, Monastir, Tunisia.

2Department of Preventive and Community Medicine, Taher Sfar University Hospital, Mahdia, Tunisia.

3Research Laboratory "Epidemiology Applied to Maternal and Child Health" 12SP17, Monastir, Tunisia.

4School of Psychology, Bond University, Australia

*Corresponding author: Emna Hariz

Department of Preventive and Community Medicine, Taher Sfar University Hospital, Mahdia, Tunisia, Research Laboratory "Epidemiology Applied to Maternal and Child Health" 12SP17, Monastir, Tunisia.

Email: emna.harizz@gmail.com

Abstract

Introduction: Stigma against people living with Human Immunodeficiency Virus (PLHIV), particularly from healthcare providers, continues to be a significant barrier discouraging individuals from seeking necessary care. However, HIV stigma, particularly among healthcare workers, is still underexplored, with limited focus on medical students. This study assesses the impact of active stigma reduction workshops on stigma towards PLHIV among a sample of medical students in Tunisia and identifies its related factors.

Method: We performed a quasi-experimental study among all fifth-year students during the academic years 2024 and 2025 in the Faculty of Medicine of Monastir (Tunisia). We evaluated HIV stigma before and after each workshop via self-administrated and anonymous questionnaire. We used the validated versions of the brief HIV knowledge questionnaire and the Health Care Provider HIV/AIDS Stigma Scale.

Results: A total of 216 medical students completely answered the questionnaire. Overall, most participants demonstrated a good level of HIV-related knowledge (88.4%). Good HIV knowledge, higher parental education and involvement in community or associative activities were significantly associated with lower stigma levels. Interestingly, using multivariate analyses, poor parental educational level and being stigmatized were independently associated with higher prejudice scores. This student-centered learning approach was revealed to be effective in significantly improving HIV-related knowledge and reducing HIV stigma among medical students.

Conclusion: Our findings emphasize the value of integrating active learning methods into the medical curriculum to prepare future healthcare providers to deliver stigma-free care in order to promote ethical and inclusive behavior toward PLHIV.

Keywords: Stigma; HIV; Medical student; Education; Tunisia.

Introduction

The Human Immunodeficiency Virus (HIV) continues to be a significant global public health challenge. In 2023, it was reported that approximately 630,000 people died from HIV-related illness worldwide (1). This disease has a significant economic effect which includes direct medical costs, lost productivity, and public health expenditures. In fact, according to the joint United Nations Program on HIV/AIDS (UNAIDS), the annual global cost of HIV/AIDS is estimated to be over $30 billion (2) . Despite major advancements in treatment and prevention, HIV continues to be a major health challenge especially in low and middle-income countries (LMIC). A study published in 2023 revealed an increasing rate in HIV incidence, mortality, and disability-adjusted life years across the Middle East and North Africa (MENA) region (3). In Tunisia, the adult HIV prevalence is estimated at 0.1%, with approximately 8,000 people living with the virus in 2022 (4).

Several factors are associated with challenges in HIV care, including limited health education, socioeconomic disparities, inadequate health literacy and persistent knowledge gap (5,6). Stigma and discrimination against people living with HIV (PLHIV) particularly from healthcare providers continues to be a significant barrier discouraging individuals from seeking necessary care (7,8). According to a qualitative study conducted in Tunisia (9), healthcare provider misconceptions about this disease are one of the major leading causes to stigmatization and thus hinder combatting the HIV epidemic. In fact, HIV discrimination manifests as reluctance to provide care to PLHIV and use of extra precautionary measures resulting in delayed diagnosis and poor treatment adherence(10). Sexual education remains a taboo and HIV infection is wrongly seen as a divine punishment and a consequence of immoral behavior especially in MENA countries (11). This knowledge gap fuels stigma both among the general population and healthcare professionals (12,13). However, HIV stigma is still underexplored in developing countries compared to developed ones, particularly among healthcare workers. For instance, a 2021 study in Sana'a, Yemen revealed discriminatory attitudes among healthcare workers stemming from a lack of institutional policies against HIV stigma (14,15).

Hence, educating healthcare providers is crucial in assessing their knowledge gap and reducing stigmatizing practices. Several studies have shown that the impact of training healthcare professionals on HIV transmission, prevention and treatment can improve their attitudes and reduce stigma(16). According to a recent study conducted among healthcare workers in Vietnam (17), focused education programs raised engagement in HIV care and decreased

stigma. It highlights the importance of educational trainings in improving healthcare worker knowledge and attitudes toward PLHIV. Addressing this issue among medical students in the medical curriculum is fundamental for establishing a generation of future healthcare professionals who can provide equitable and compassionate care to PLHIV. Indeed, medical student attitudes and knowledge about HIV are shaped during their training, making this population a key target for interventions aimed at reducing stigma(18). However, most research on HIV-related stigma was among practicing clinicians, with limited focus on medical students, particularly in LMICs. A recent study in Saudi Arabia assessed this gap, reporting misconceptions and poor HIV knowledge among medical students. Thus, it is important to establish educational interventions among pre-clinical medical students.

Furthermore, while educational interventions have shown significant effects in reducing stigma, the effectiveness of active learning methods remains underexplored in the context of HIV (19). For instance, a thematic review revealed that the student-centered learning (SCL) method has the ability to foster deeper engagement, empathy and understanding by placing learners at the center of the educational process preparing them for professional challenges (20).

Given these data, the current study aimed to assess the impact of active training (workshops) on reducing stigma against PLHIV among fifth medical students in the Faculty of Medicine of Monastir (Tunisia) and to explore its related factors.

Method

Sampling and data collection:

We performed a quasi-experimental study during the academic years 2023–2024 and 2024–2025 among all fifth-year students in the Faculty of Medicine of Monastir (Tunisia), who have one remaining year of clinical training at the hospital during which they are responsible for patient care. The recruitment period for the first academic year was from 15/09/2023 to 31/05/2024, and for the second academic year from 15/09/2024 to 31/05/2025. The total duration of teaching during the academic year was 10 months.

A total of 205 students participated in our study, following the precedent set by a similar study by Al-Fadhli et al. (21). Their survey assessed the impact of HIV stigma-reduction workshops on healthcare providers in two tertiary-care hospitals and three healthcare colleges in central India, including 650 participants, of whom 205 were medical students (21). Despite different contexts, the intervention and population are similar, making it a relevant sample size reference.

Every week, a 2-hour workshop about HIV knowledge and practices was conducted for groups of 10 medical students at the Department of Preventive and Community Medicine within the faculty of Medicine of Monastir. The teacher, a professor of medicine, facilitated the sessions based on presentations and interactive lectures.

The intervention consisted of animating these workshops based on the SCL approach (20). This method shifts the traditional focus from instructor-led teaching to one that places students at the heart of the learning experience. It focuses on courses and classroom-based role-plays lectures, and open discussions prepared and delivered actively by students.

We evaluated HIV knowledge and practices as well as stigma attitudes towards PLHIV before and after each workshop using self-administered and validated measurement tools.

Measurement tools:

The study instrument in the current study was a self-administered questionnaire, and consisted of three sections as follows:

- **Knowledge about HIV among medical students**

To assess HIV knowledge, we used the validated French version of the **brief HIV knowledge questionnaire** (HIV-KQ-18). This questionnaire was originally developed by Carey et al. (2004) and was later validated in French (22). This tool is self-administered, which is designed

to assess general knowledge about HIV among medical students, modes of transmission, prevention methods and common misconceptions of the disease. It includes 18 yes or no statements evaluating attitudes and practices. Only five of the items are true (items 1, 4, 11, 14 and 17). The correct response is coded one, whereas the incorrect answers receive zero points with a maximum score obtainable for knowledge being 18 marks. Having a score of 14 or higher indicates a good HIV knowledge level, whereas a score below 14 signifies poor knowledge (23).

Stigma attitudes towards PLHIV among medical students

To evaluate stigma attitudes towards PLHIV, we used the validated instrument "**Health Care Provider HIV/AIDS Stigma Scale** (HPASS)" (24). The HPASS consists of a 30-item structured questionnaire. Each item is an example of a scenario that physicians might encounter in their future professional life or certain beliefs regarding PLHIV. In each statement, responders choose from six possible answers ‘’ strongly disagree, ‘’ ‘’slightly disagree ‘’ disagree’’ ‘’agree’’, ‘’slightly agree’’ or ‘’ strongly agree’’. These responses are assigned corresponding scores of 0, 1, 2, 3, 4, 5, respectively. This tool evaluates three dimensions (25): prejudice, stereotyping and discrimination, divided as follows:

- **Prejudice** (Items 1 to 13): These items reflect emotional reactions and negative attitudes towards PLHIV, including feelings of fear, blame or discomfort.
  A high score in this domain represents a stronger prejudicial attitude.
- **Stereotyping** (Items 14 to 23): These items evaluate negative beliefs towards PLHIV; assumptions about their behavior, lifestyle, or character. Stereotyping reflects cognitive biases often rooted in misinformation or cultural stigma
- **Discrimination** (Items 24 to 30): These items refer to discriminatory behavior or intentions, such as avoidance or unequal treatment of PLHIV. High scores indicate a greater likelihood of engaging in discriminatory actions.

In this section, we did not use predefined cut-off values to categorize participants into "low" or "high" stigma groups. Instead, the total score presented by the cumulative level of stigma, was considered as a continuous measure, with higher scores reflecting greater levels of stigma whether in the form of prejudice, stereotyping, or discriminatory behaviors. Comparing the mean scores before and after the intervention allows us to identify patterns or shifts in stigma and thus evaluate the impact of the active learning method (26).

- **Sociodemographic characteristics and sexual practices of participants.**

We collected data about sociodemographic characteristics and sexual practices to assess the potential influence of these factors on HIV related stigma. These questions were added based on a literature review about factors associated with HIV stigma. In the sociodemographic characteristics section, the collected data comprises: age, gender and parental educational level. Parental educational level was recorded into two categories: High educational level (at least one parent has completed higher education) and poor educational level (both parents have education levels below high school). We also asked about involvement in associative activities, HIV unit or screening campaigns and having received HIV-related training. The source of information on sexual and reproductive practices was grouped into reliable sources (Parents, Siblings, Cousins, School/University) and unreliable sources (Friends, Media, Internet). Data about experiencing stigma and sexual practices including: Previous sexual experience, condom protection, substance use during sex and sexually transmitted infections (STI) screening were collected.

Cultural context: Tunisia is a Muslim-majority country, and topics such as sexual behavior and HIV are often socially sensitive or somewhat taboo. In this context, HIV is generally associated with high-risk sexual practices, which may influence participants' willingness to report certain behaviors or attitudes honestly. This consideration was taken into account when designing the questionnaire and interpreting the results.

Statistics analysis:

Data entry and analysis were conducted using the Statistical Package for social science (SPSS); Version 23.0. Qualitative variables were represented by effectives and percentages. After normal distribution testing, continuous variables were expressed as the mean plus or minus the standard deviation (mean ± SD).

To compare variables with HIV knowledge levels, we used the Chi 2 test ($\chi 2$) or Fisher exact test since we compared percentages. To determine factors associated with stigma score and its three dimensions, we performed a univariate analysis using the student test to compare two independent means. The multivariate analysis via the linear regression model was conducted to identify independent factors associated with stigma scores.

Regarding the evaluation of the impact of the active workshop, dependent sample t-tests were performed to compare the mean scores of HIV knowledge and stigma before and after the active training method at the significant level of 0.05. Also, we calculated the Relative Reduction

(Percentage Change): as the difference between post-intervention and pre-intervention means, divided by the pre-intervention mean, and multiplied by 100.

Ethical considerations:

Participation in our study was voluntary. All participants provided informed consent and the study was anonymous. Students responded to the questionnaire in separate and distant tables. The data obtained from the students was solely used for the study purpose and was not included in the final score of the exam. The authors had no access to information that could identify students after data collection. The Ethic Committee of the University Hospital in Mahdia (CEM 2024-02-08) approved the study protocol.

Results

Sociodemographic characteristics and sexual practices of participants:

A total of 216 medical students answered the questionnaire completely with a response rate of 43.2%. The mean age of participants was 23 years±0.84 with a majority of female (63%). Participant background demographics and practices are outlined in Table 1.

Table 1: Sociodemographic characteristics and sexual practices among medical students.

Variables	Categories	Effective	Percentage
**Educational level of parents (N=216)**	High	157	72.7%
	Poor	59	27.3%
**Involvement in associative activities, HIV unit or screening campaigns (N=216)**	Yes	50	23.1%
	No	166	76.9%
**Source of sexual knowledge (N=216)**	Reliable	81	37.5%
	Unreliable	135	62.5%
**Experiencing stigma or discrimination (N=216)**	Yes	31	14.4%
	No	185	85.6%
**Previous sexual experience (N=216)**	Yes	28	13%
	No	188	87%
**Condom protection (N=28)**	Yes	16	57.1%
	No	12	42.9%
**Substance use during sex (N=28)**	Yes	24	50%
	No	24	50%
**STI screening (N=216)**	Yes	5	2.3%
	No	211	97.7%

Pre-intervention HIV stigma among medical students and its related factors:

The majority of participants presented good knowledge levels (88.4%). Table 2 presents the results of univariate analysis on variables influencing the total stigma score among medical students. In fact, individuals having good HIV knowledge, high parental educational level and those who were involved in associative activities, HIV units or screening campaigns exhibited lower stigma scores (Table 2).

Table 2: Factors influencing the total stigma score among medical students: Univariate analysis

Variables	Categories	Mean stigma score±SD	P value
**HIV knowledge (N=216)**	Good	**59.34±22.2**	**0.01**
	Poor	**69.88±17.63**	
**Gender (N=216)**	Male	59.76±24.65	0.685
	Female	61.02±20.31	
**Educational level of parents (N=216)**	High	**58.65±22.36**	**0.037**
	Poor	**65.64±20.12**	
**Involvement in associative life HIV unit or screening campaigns (N=216)**	Yes	**53.14±22.07**	**0.006**
	No	**62.79±21.47**	
**Source of sexual knowledge (N=216)**	Reliable	60.26±23.67	0.876
	Unreliable	60.74±20.93	
**Experiencing stigma or discrimination (N=216)**	Yes	64.48±24.11	0.283
	No	60±21.56	
**Previous Sexual experience (N=216)**	Yes	61.43±21.04	0.213
	No	54.67±26.96	
**Condom protection (N=28)**	Yes	46.56±25.96	0.065

	No	65.5±25.33	
**Substance use during sex (N=28)**	Yes	55.54±28.17	0.576
	No	59.46±19.2	
**STI screening (N=216)**	Yes	50.4±20.41	0.296
	No	60.8±22	

Prejudice related factors

Table 3 highlights variables influencing prejudice among dimension among medical students through univariate analysis. Having a high educational level of parents and being involved in associative activities, HIV units or screening campaigns were associated with lower prejudice scores (Table 3).

Table 3: Factors influencing prejudice dimension among medical students: Univariate analysis

**Variables**	**Categories**	**Mean prejudice score±SD**	**P value**
**HIV knowledge (N=216)**	Good	26.68±11.47	0.094
	Poor	30.72±9.78	
**Gender (N=216)**	Male	26.44±12.684	0.489
	Female	27.55±10.51	
**Educational level of parents (N=216)**	High	**25.97±11.25**	**0.013**
	Poor	**30.27±11.07**	
**Involvement in associative activities, HIV unit or screening campaigns (N=216)**	Yes	**22.7±11.23**	**0.001**
	No	**28.49±11.06**	
**Source of sexual knowledge(N=216)**	Reliable	26.65±12.43	0.621
	Unreliable	27.44±10.67	

**Experiencing stigma or discrimination (N=216)**	yes	**30.93±13.74**	**0.044**
	No	**26.51±10.8**	
**Previous Sexual experience (N=216)**	Yes	25.28±13.66	0.353
	No	27.42±10.97	
**Condom protection (N=28)**	Yes	21.93±11.9	0.137
	No	29.75±15.06	
**Substance use during sex (N=28)**	Yes	26.04±13.86	0.972
	No	26.16±10.15	
**STI screening (N=216)**	Yes	25.2±10.1	0.699
	No	27.19±11.38	

Multivariate linear regression showed that lower parental educational level and experiences of stigma or discrimination were independently associated with higher HIV prejudice scores (for more details, see table4).

Table 4: Factors influencing prejudice among medical students: Multivariate linear regression analysis

Related Factors to HIV prejudice	Standard Errors	P value	B coefficient	IC95%
**Educational level of parents (N=216)**	5.033	0.045	0.359	0.27 - 21.05
**Experiencing stigma or discrimination (N=216)**	5.925	0.042	0.364	0.53 - 24.98

Stereotype related factors:

Table 5 shows variables influencing stereotype among medical students through univariate analysis.

Table 5: Factors influencing stereotype among medical students: Univariate analysis

Variables	Categories	Mean stereotype score±SD	P value

**HIV knowledge (N=216)**	Good	21.79±8.15	0.166
	Poor	24.2±8	
**Educational level of parents (N=216)**	High	**21.34±8.2**	**0.033**
	Poor	**24±7.8**	
**Involvement in associative life HIV unit or screening campaigns (N=216)**	Yes	22.54±8.01	0.121
	No	20.5±8.52	
**Previous Sexual experience (N=216)**	Yes	19.67±10.1	0.097
	No	22.42±7.8	
**Condom protection (N=28)**	Yes	**16.31±10.34**	**0.039**
	No	**24.16±8.13**	

Discrimination dimension:

No variables influencing the discrimination dimension among medical students were found through univariate analysis in our sample.

Impact of SCL method on HIV knowledge and stigma against PLHIV:

Table 6 shows the score trends before and after the active workshops (knowledge, stigma, prejudice, stereotype and discrimination scores).

Regarding HIV knowledge, the overall score increased significantly from 15.57±1.61 to 16.3±1.5. A notable reduction was observed in the stigma score following the SCL method. In fact, the mean score significantly decreased from 60.56±21.94 to 52.64±22. Similar results were found for all stigma dimensions (Table 6).

Table 6: Impact of SCL method on HIV knowledge and stigma dimensions (N=216)

Variable	Mean knowledge and stigma scores ±SD		P value	Relative change (%)
	Before	After		
**Knowledge score**	**15.57±1.61**	**16.3±1.5**	**<0.001**	**+4.69**
**Total Stigma score**	**60.56±21.94**	**52.64±22**	**<0.001**	**-13.08**
**Prejudice score**	**27.14±11.34**	**22.57±11.09**	**<0.001**	**-16.84**
**Stereotype score**	**22.07±8.16**	**20.45±8.51**	**<0.001**	**-7.34**
**Discrimination score**	**11.34±7.54**	**9.61±7.29**	**<0.001**	**-15.26**

Discussion:

To our knowledge, our study is among the first in North Africa to focus on stigma among medical students against PLHIV and to assess the impact of the SCL method on both knowledge and stigma. In fact, we found that of all stigma dimensions, prejudice showed significant associations in multivariate analysis. Interestingly, poor parental educational level and being stigmatized were independently associated with higher prejudice scores. In addition, we found that the SCL method was effective in significantly improving HIV-related knowledge as well as in reducing stigma scores among medical students.

We found that the majority of participants were not involved in HIV related activities or screening campaigns (76.9%). This result is in contrast to findings in Hong Kong showing that 72.1% of final-year medical students were exposed to HIV patients during attachment to an HIV clinic in the late 2000s, compared with 39.3% in the mid-2010s (27). The significantly lower exposure in Tunisia (23.1%) compared to Hong Kong likely stems from structural and institutional differences. Tunisian HIV care remains highly centralized in a few specialized units. Furthermore, pervasive socio-cultural stigma in Tunisia often necessitates a higher degree of patient anonymity, which, combined with strict confidentiality protocols, limits the integration of HIV-positive patients into general undergraduate clinical training. More broadly, in MENA countries, stigma, cultural taboos and lack of structured programs may contribute to the low involvement rates (11). In our study, most medical students reported having an unreliable source of sexual knowledge (85.5%). However, studies in Iran and Greece reported greater use of reliable source (28). This may be influenced by cultural and societal differences. In fact, in Tunisia, sexual health topics are still taboos leading younger individuals to seek false information elsewhere.

Among students who were sexually active in our sample, only 57.1% use condoms for protection. This contrasts with results among Mexican medical students in 2024, where a higher proportion of students reported condom use during vaginal intercourse (83.6 % in men and 67.7% in women) (29). However, condom use cannot be attributed only to the level of sexual health education. Indeed, other influencing factors such as partner preference, access to condoms, personal and cultural attitudes affect the use of this protective measure (30). We found that only 2.3% of participants underwent STI screening. Similarly, in a 2023 study conducted in Warsaw, Poland, Only 21.43% of participants had ever been tested for HIV (31). Several explanations could be cited such as low sexual encounters, fear of being stigmatized for testing and lack of HIV related knowledge (31). In our cultural context, sexual health remains a

sensitive topic, which may explain the very low STI screening rates and reliance on unreliable sources of information. These findings highlight the urgent need for broader, culturally appropriate sexual health education among medical students.

As for HIV knowledge, findings in our study showed a good knowledge level among 88.4% of medical students. In fact, in the Tunisian medical curriculum, students start learning about HIV and its impact early in their education. This is in accordance with results reported in Iran and in countries from both sub-Saharan Africa and North Africa (32,33). Nevertheless, it remains essential to sustain efforts aimed at further enhancing HIV knowledge by screening the related factors and by developing targeted interventions in educational medical trainings. To the best of our knowledge, our study is the first to focus on stigmatizing attitudes towards PLHIV among medical students in Tunisia. As medical students play a key role in future care delivery, it is crucial to assess stigma and its related factors. Students with higher scores on HIV-related knowledge exhibit lower stigma rates. These results align with studies conducted in Yemen and Indonesia among healthcare professionals, where poor knowledge of HIV was linked to higher rates of stigma (34,35). However, an Indonesian study suggested that physicians exhibited both the highest HIV knowledge and the highest levels of stigma (36). Thus, combatting stigma requires interventions that transcend knowledge and highlight cultural beliefs and other factors influencing healthcare professionals' attitudes. We found that female participants exhibit a higher stigma score compared to males. These results align with findings from an Iranian study (37) and contradicts with another study from the USA (38). However, the gender difference observed in our study was not statistically significant and should therefore be interpreted with caution. In certain sociocultural contexts where discussions surrounding sexuality and HIV may be more socially constrained for women, gender-related norms could potentially influence attitudes. This hypothesis warrants further investigation in larger and more diverse samples. In our survey, students who were involved in associative activities, HIV units and screening campaigns exhibited a lower stigma score. Likewise, a study conducted in Jordan among medical students stated that pre-clinical students had a significantly more negative attitude towards PLHIV compared to their clinical counterparts (39). Likewise, from a study carried out among students from Hong Kong, it was found that pupils who were not willing to provide care for PLHIV had less clinical experience with HIV patients (27). We found that individuals untested for HIV tend to have a higher stigma score. These findings align with results from Indonesia (35). One possible explanation is that during HIV testing and counseling, individuals might gain great HIV knowledge and undergo the emotional experience of diagnosis. Our study

found no significant association between the reliability of the source and stigma rates. However, although the effect is weak, the media was found to be an enhancing factor for HIV knowledge and a reducing factor of HIV stigma in Iran (40). The media is a powerful tool for combatting stigma against PLHIV when it is guided by accurate information about the disease. However, in many conservative societies, PLHIV continue to face negative representations in the media, reinforcing panic and misconceptions. Thus, it is urgent to implement media-based awareness and evidence-based education(41).

HIV prejudice refers to negative feelings, judgments, and attitudes towards PLHIV rooted in negative beliefs and often reinforced by fear of the disease (25). We found that students with better HIV-related knowledge showed lower levels of prejudice. Similarly, dental healthcare providers in Pakistan with limited HIV-related knowledge tended to hold more negative attitudes towards HIV (42). Participation in HIV-related workshops or screening campaigns was linked to a lower prejudice score in our sample. Likewise, trainings on HIV stigma and/or discrimination was associated with less negative attitudes in healthcare providers in Germany (43). This highlights the importance of structured educational interventions for reducing prejudice among healthcare professionals. Beyond the univariate analysis, multivariate analysis of stigma dimensions revealed specific predictors of prejudice. Notably, previous personal experience of being stigmatized and poor parental educational level were independently associated with higher prejudice scores. Limited research has explored how an individual's own history of being stigmatized shapes their attitudes toward PLHIV. However, based on results reported in Zambia, healthcare professionals who did not stigmatize but witnessed stigmatizing attitudes among their coworkers, were more likely to hold critical and stigmatizing attitudes (44). We did not find evidence of an association between the parental educational level of students and their attitude towards PLHIV. On the other hand, in Indonesia, belonging to the richest or rich economic group was associated with higher levels of stigma against PLHIV (45). In contrast, a Ugandan study reported that wealthier individuals were less likely to endorse social distance and stigma against PLHIV(46). These findings suggest that neither wealth nor parental education level predicts young people's attitudes. To the best of our knowledge, this is the first study to report associated factors with prejudice scores. Nonetheless, factors such as relationship dynamics, personal convictions, and social barriers must also be taken into account when evaluating attitudes toward HIV. Assessing related factors to HIV stigma into medical curriculum is needed to prepare future professionals into delivering compassionate and equitable care to PLHIV and to combat HIV decease (47).

Reducing stigma is essential to improving healthcare outcomes and care delivery especially among healthcare professionals (17,48). Through the years, Depersonalization of patient cases and lack of diverse learning methods have limited student engagement and empathy. Traditional learning approaches often fail to nurture humanistic and relational skills. As a result, various learning methods have been used in different curricula and medical education such as problem-based learning, Case-based discussions, and flipped-classroom methods. Another innovative approaches involve Game-based learning, Team-based learning , Simulation-based learning, and peer teaching. They all embody the concept of SCL placing the learner at the core of the educational process (49). It involves active participation where students engage in role-playing scenarios, open discussions and lectures. Students take on the roles of actors in various role-playing scenarios and are often placed in patient and doctor shoes (50). Further, a range of educational interventions has been employed for HIV-stigma reduction (16). Thus, we aimed to assess the impact and effectiveness of this method in enhancing HIV-related knowledge and reducing stigma among medical students. In our study, after the SCL approach, the overall HIV knowledge score significantly improved. A significant decline in HIV stigma scores was also noted and similar improvements were observed across all stigma dimensions. For instance, similar Tunisian data showed that both active participants and observers achieved significant gains in knowledge scores after a role-play simulation (51). Similarly, a Tunisian study assessed the impact of the SCL among medical students in the faculty of medicine of Monastir. Significant improvement in hand hygiene and biomedical waste management after the SCL intervention (52).

Beyond its implication in general medical subjects, the SCL method has also been explored in our primary focus, enhancing HIV-related knowledge and reducing stigma against PLHIV. Given the HPASS scoring range, the observed 7.9-point decrease reflects a tangible reduction in stigmatizing attitudes, indicating that the intervention may have practical educational relevance in shaping future healthcare professionals' perspectives toward PLHIV. For instance, a peer-led education intervention among secondary school students in Sudan showed a significant improvement in participant knowledge, attitudes and practices regarding HIV. This study highlights the effectiveness of SCL and peer-based approaches in promoting HIV awareness and reducing HIV stigma among medical students (53). Similarly, a pilot study in the United States assessed the Facilitated Resilience and Equity through Stigma-reduction and Health workshop, a reduction of HIV-related stigma intervention among healthcare workers. During the workshop, participants interacted through discussions and role-playing. Findings

showed a significant reduction in stigma and a significant HIV-related knowledge increase (54). In addition, in China, a web-based game was developed to reduce HIV-related stigma. The game used participatory gamification and storytelling to immerse university students in the lives of PLHIV. This approach enhanced empathy-building, active learning through problem solving and reflection (55). In addition, our study results align with a previous Indian study conducted among healthcare professionals (56). The HIV-related stigma reduction workshops in Central India significantly impacted the knowledge and attitudes of healthcare providers and students. These results were assessed through a pre and post-test similar to our intervention.

To our knowledge, our study is the first globally and in the MENA region to simultaneously integrate the HIV-KQ-18 and the HPASS in a single research framework among medical students. HIV knowledge and stigma are critical issues in the MENA region, where persistent misconceptions, cultural taboos, and deeply rooted stigma continue to hinder efforts in HIV prevention and patient care. Addressing these challenges among medical students as future healthcare providers is an essential urgent step toward a long-term stigma-free environment and improved quality of life for PLHIV. It is essential to form medical graduates with a solid foundation in professional ethics, empathy, and a clear understanding of patient rights and confidentiality, ensuring they are prepared to deliver respectful and stigma-free care for patients. Uniquely, our study also incorporates a before-and-after intervention design, embedding active training and SCL modules within the medical education curriculum.

These results should be considered in light of the study limitations. First, it was a single-center study with a limited sample size. Since the study was conducted among medical students from one institute, findings cannot be generalized to broader populations of medical or healthcare students. Similar constraints were observed in a Vietnamese study among medical students who assessed HIV knowledge and stigma among a limited group of 200 medical students (57). In addition, the Faculty of Medicine of Monastir operates within a specific educational, institutional, and sociocultural context that may influence students' attitudes toward PLHIV. Tunisia is part of the MENA region, where HIV-related stigma is often shaped by religious norms, cultural perceptions surrounding sexuality, and societal attitudes toward key populations. These contextual factors may influence baseline stigma levels as well as responsiveness to educational interventions. Furthermore, variations in curriculum content, clinical exposure to PLHIV, and institutional culture across medical faculties may affect the transferability of our findings to other settings, both nationally and internationally. To enhance the generalizability and reliability of the findings, it is recommended to generalize our

intervention to all healthcare institutes on a national scale for reliable results. Secondly, one important limitation of this study was the lack of future follow-up of the impact of the intervention. While the training aimed to reduce HIV stigmatizing attitudes among medical students, changes in practices among healthcare professionals were not evaluated. The current research did not conduct long-term follow-up surveys to verify the sustainability of the training, making it impossible to determine whether the training truly had a positive impact on the participant behavior against PLHIV. This gap between theoretical training and healthcare practices is highlighted in an Indian study, which emphasized the absence of stage-based follow-up to assess the effectiveness of the reduction workshops (56). Future studies should integrate multiple follow-up stages for a deeper understanding of how knowledge and attitudes evolve and potentially translate into lasting behavioral change. Furthermore, because stigma was measured immediately after the intervention, we cannot determine whether the observed reduction reflects a sustained change in attitudes or a short-term response. Future research is planned to include longitudinal follow-up assessments at multiple time points to evaluate the durability of attitude change over time. Thirdly, in our study, the relatively low response rate (43.2%) may reflect potential bias, as some students may have chosen not to participate due to the sensitive nature of certain questions related to sexuality and religious or cultural norms.

Conclusion

Our findings underscore the urgent need to integrate continuous anti-stigma education into medical curricula through active learning approaches. Interventions must be introduced early in the medical curriculum and backed by strong institutional commitment. Moreover, such efforts must be sustained and extend beyond healthcare settings to target the general population fostering an informed and nondiscriminatory society. Within the healthcare environment and the general population, a graduated accountability system should be introduced to address stigma early on.

Acknowledgements:

We would like to thank all the participants of the study.

Data Availability Statement:

All data underlying the findings described in this manuscript are fully available within the Supporting Information file. The Excel dataset (S1_Data.csv) can be freely accessed.

References

1. World Health Organisation. HIV [Internet]. 2025 [cited 2025 Feb 3]. Available from: https://www.who.int/data/gho/data/themes/hiv-aids?utm_source=chatgpt.com

2. UNAIDS. Global HIV & AIDS statistics — Fact sheet [Internet]. 2025 [cited 2025 Feb 3]. Available from: https://www.unaids.org/en/resources/fact-sheet

3. Khorrami Z, Balooch Hasankhani M, Khezri M, Jafari-Khounigh A, Jahani Y, Sharifi H. Trends and projection of incidence, mortality, and disability-adjusted life years of HIV in the Middle East and North Africa (1990–2030). Sci Rep. 2023 Aug 24;13(1):13859.

4. UNAIDS. Tunisia [Internet]. 2025 [cited 2025 Feb 5]. Available from: https://www.unaids.org/en/regionscountries/countries/tunisia

5. Kumah E, Boakye DS, Boateng R, Agyei E. Advancing the Global Fight Against HIV/Aids: Strategies, Barriers, and the Road to Eradication. Ann Glob Health [Internet]. [cited 2025 May 13];89(1):83.

6. Bouabida K, Chaves BG, Anane E. Challenges and barriers to HIV care engagement and care cascade: viewpoint. Front Reprod Health [Internet]. 2023 Jul 20 [cited 2025 May 13];5.

7. European Centre for Disease Prevention and Control. HIV stigma in healthcare settings: need for increased knowledge among healthcare workers and improved facility-level guidelines. Stockholm: ECDC; 2024 [cited 2025 May 13]. Available from: https://www.ecdc.europa.eu/en/news-events/hiv-stigma-healthcare-settings-need-increased-knowledge-among-healthcare-workers-and

8. Crockett KB, Turan B, Whitfield S, Kay ES, Budhwani H, Fifolt M, et al. Patient and Provider Perspectives on HIV Stigma in Healthcare Settings in Underserved Areas of the US South: A Mixed Methods Study. AIDS Behav [Internet]. 2022 Jan [cited 2025 May 13];26(Suppl 1):112–24.

9. Zribi M, Mansour NB, Moussa H, Hassine HB, Aounallah-Skhiri H. Experiences and perceptions of health professionals towards the quality of care for people living with HIV in Tunisia: a qualitative study. Pan Afr Med J [Internet]. 2023 Sep 6 [cited 2025 Feb 5];46:4.

10. Armoon B, Fleury MJ, Bayat AH, Fakhri Y, Higgs P, Moghaddam LF, et al. HIV related stigma associated with social support, alcohol use disorders, depression, anxiety, and suicidal ideation among people living with HIV: a systematic review and meta-analysis. Int J Ment Health Syst [Internet]. 2022 Mar 4 [cited 2025 Feb 3];16(1):17.

11. Kteily-Hawa R, Hawa AC, Gogolishvili D, Al Akel M, Andruszkiewicz N, Vijayanathan H, et al. Understanding the epidemiological HIV risk factors and underlying risk context for youth residing in or originating from the Middle East and North Africa (MENA) region: A scoping review of the literature. PLoS ONE [Internet]. 2022 Jan 7 [cited 2025 Apr 13];17(1):e0260935.

12. Aldhaleei WA, Bhagavathula AS. HIV/AIDS-knowledge and attitudes in the Arabian Peninsula: A systematic review and meta-analysis. J Infect Public Health [Internet]. 2020 Jul 1 [cited 2025 May 13];13(7):939–48.

13. Elghazaly A, AlSaeed N, Islam S, Alsharif I, Alharbi L, Al Ashagr T, et al. Assessing the knowledge and attitude towards HIV/AIDS among the general population and health care professionals in MENA region. PLOS ONE [Internet]. 2023 Jul 27 [cited 2025 May 13];18(7):e0288838.

14. Attal BA, Al-Rowaishan KM, Akeel AA, AlAmmar FK. HIV stigma in the teaching hospitals in Sana'a, Yemen: a conflict and low-resource setting. BMC Public Health [Internet]. 2021 Oct 5 [cited 2025 Apr 18];21:1793.

15. Rizk NA, Helou RE, AlMukdad S, Fahme S, Mumtaz GR, Chemaitelly H, et al. Has UNAIDS left the Middle East and north Africa behind? Lancet HIV [Internet]. 2023 Oct 1 [cited 2025 May 13];10(10):e630–2.

16. Ferguson L, Gruskin S, Bolshakova M, Rozelle M, Yagyu S, Kasoka K, et al. Systematic review and quantitative and qualitative comparative analysis of interventions to address HIV-related stigma and discrimination. AIDS Lond Engl [Internet]. 2023 Nov 1 [cited 2025 May 1];37(13):1919–39.

17. Pollack TM, Duong HT, Nhat Vinh DT, Phuong DT, Thuy DH, Nhung VTT, et al. A pretest-posttest design to assess the effectiveness of an intervention to reduce HIV-related stigma and discrimination in healthcare settings in Vietnam. J Int AIDS Soc. 2022 Jul;25 Suppl 1(Suppl 1):e25932.

18. Alshouibi E, Alaqil F. HIV-Related Discrimination among Senior Dental Students in Jeddah. J Int Soc Prev Community Dent [Internet]. 2019 [cited 2025 May 13];9(3):219–24.

19. Taggart T, Taboada A, Stein JA, Milburn NG, Gere D, Lightfoot AF. AMP!: A Cross-site Analysis of the Effects of a Theater-based Intervention on Adolescent Awareness, Attitudes, and Knowledge about HIV. Prev Sci Off J Soc Prev Res [Internet]. 2016 Jul [cited 2025 May 13];17(5):544–53.

20. (PDF) A thematic review on student-centred learning in the studio education [Internet]. [cited 2025 Feb 6]. Available from: https://www.researchgate.net/publication/339640951_A_thematic_review_on_student-centred_learning_in_the_studio_education?utm_source=chatgpt.com

21. Machowska A, Bamboria BL, Bercan C, Sharma M. Impact of 'HIV-related stigma-reduction workshops' on knowledge and attitude of healthcare providers and students in Central India: a pre-test and post-test intervention study. BMJ Open [Internet]. 2020 Apr 1 [cited 2025 Jan 24];10(4):e033612.

22. Carey MP, Schroder KEE. Development and Psychometric Evaluation of the Brief HIV Knowledge Questionnaire. AIDS Educ Prev Off Publ Int Soc AIDS Educ [Internet]. 2002 Apr [cited 2025 Feb 7];14(2):172–82.

23. Wagenaar BH, Sullivan PS, Stephenson R. HIV Knowledge and Associated Factors among Internet-Using Men Who Have Sex with Men (MSM) in South Africa and the United States. PLoS ONE [Internet]. 2012 Mar 12 [cited 2025 Jan 25];7(3):e32915.

24. Wagner AC, Hart TA, McShane KE, Margolese S, Girard TA. Health Care Provider Attitudes and Beliefs About People Living with HIV: Initial Validation of the Health Care Provider HIV/AIDS Stigma Scale (HPASS). AIDS Behav [Internet]. 2014 Dec 1 [cited 2025 Jan 24];18(12):2397–408.

25. Wagner AC, Hart TA, McShane KE, Margolese S, Girard TA. Health Care Provider Attitudes and Beliefs About People Living with HIV: Initial Validation of the Health Care Provider HIV/AIDS Stigma Scale (HPASS). AIDS Behav [Internet]. 2014 Dec 1 [cited 2025 Jan 24];18(12):2397–408.

26. Heidarpoor P, Mousavikashi S. Effectiveness of peer assisted learning in improving HIV knowledge, attitude, and perception among community health workers. Res Dev Med Educ [Internet]. 2023 Aug 29 [cited 2025 Jan 25];12(1):6–6.

27. HKMJ [Internet]. 2022 [cited 2025 Apr 13]. Serial surveys of Hong Kong medical students regarding attitudes towards HIV/AIDS from 2007 to 2017.

28. Lagadinou M, Spiliopoulou K, Paraskevas T, Gkentzi D, Assimakopoulos S, Katsakiori P, et al. Knowledge and Attitudes of Medical and Nursing Students in a Greek University Regarding Sexually Transmitted Diseases. Int J Environ Res Public Health [Internet]. 2024 Feb 22 [cited 2025 Apr 12];21(3):251.

29. Garcia-Romo GS, Pozo-Molina G, Reyes-Reali J, Mendez-Catala CF, Garrido E, Mendez-Cruz AR, et al. Prevalence of sexually risky behaviors among Mexican medical students. PLOS ONE [Internet]. 2024 May 6 [cited 2025 Apr 12];19(5):e0302570.

30. Lalo R, Theodhosi G, Breshanaj A. Health beliefs and barriers related to HIV prevention and screening among students of the University of Vlora: a cross-sectional study. BMC Public Health [Internet]. 2020 Aug 27 [cited 2025 Apr 14];20:1302.

31. Kowalska J, Cholewik M, Bieńkowski C, Maciejczyk A, Bursa D, Skrzat-Klapaczyńska A. Knowledge and Awareness of Risk Factors for HIV Infection and about HIV Testing among Medical Students in Warsaw. Viruses [Internet]. 2024 Sep 15 [cited 2025 Apr 12];16(9):1470.

32. Pourjam R, Rahimi Khalifeh Kandi Z, Estebsari F, Karimi Yeganeh F, Safari M, Barati M, et al. An Analytical Comparison of Knowledge, Attitudes, and Practices Regarding HIV/AIDS Among Medical and Non-Medical Students in Iran. HIVAIDS Auckl NZ [Internet]. 2020 Apr 28 [cited 2025 Apr 12];12:165–73.

33. Terra M, Okereke PU, Wandera F, Edith K, Kitonga MS, Ally AM, et al. HIV related knowledge and practices among undergraduate students in Africa: A cross-sectional multinational study. J Med Surg Public Health [Internet]. 2024 Aug 1 [cited 2025 Apr 16];3:100126.

34. Attal BA, Al-Rowaishan KM, Akeel AA, AlAmmar FK. HIV stigma in the teaching hospitals in Sana'a, Yemen: a conflict and low-resource setting. BMC Public Health [Internet]. 2021 Oct 5 [cited 2025 Jul 27];21:1793.

35. Fauk NK, Ward PR, Hawke K, Mwanri L. HIV Stigma and Discrimination: Perspectives and Personal Experiences of Healthcare Providers in Yogyakarta and Belu, Indonesia. Front Med [Internet]. 2021 May 12 [cited 2025 Apr 18];8:625787.

36. Hidayat J, Chen MY, Maulina R, Nurbaya S. Factors Associated With HIV-Related Stigma Among Indonesian Healthcare Workers: A Cross-Sectional Online Survey. J Nurs Res [Internet]. 2023 Oct [cited 2025 Apr 20];31(5):e295.

37. Tavakoli F, Karamouzian M, Rafiei-Rad AA, Iranpour A, Farrokhnia M, Noroozi M, et al. HIV-Related Stigma Among Healthcare Providers in Different Healthcare Settings: A Cross-Sectional Study in Kerman, Iran. Int J Health Policy Manag [Internet]. 2019 Oct 21 [cited 2025 Apr 18];9(4):163–9.

38. Stringer KL, Turan B, McCormick L, Durojaiye M, Nyblade L, Kempf MC, et al. HIV-Related Stigma among Healthcare Providers in the Deep South. AIDS Behav [Internet]. 2016 Jan [cited 2025 Apr 18];20(1):115–25.

39. Sallam M, Alabbadi AM, Abdel-Razeq S, Battah K, Malkawi L, Al-Abbadi MA, et al. HIV Knowledge and Stigmatizing Attitude towards People Living with HIV/AIDS among Medical Students in Jordan. Int J Environ Res Public Health [Internet]. 2022 Jan 10 [cited 2025 Apr 15];19(2):745.

40. Aghaei A, Mohraz M, Shamshirband S. Effects of media, interpersonal communication and religious attitudes on HIV-related stigma in Tehran, Iran. Inform Med Unlocked [Internet]. 2020 Jan 1 [cited 2025 Apr 20];18:100291.

41. Iacoella F, Gassmann F, Tirivayi N. Impact of mobile phones on HIV public stigma: a cross-sectional and pseudo-panel analysis from Ghana. BMJ Open [Internet]. 2022 Nov 8 [cited 2025 May 2];12(11):e062594.

42. Rana BK, Sarfraz M, Reza TE, Emmanuel F. A Cross-Sectional Study to Assess HIV/AIDS-Related Stigma and Its Drivers Among Dental Healthcare Providers in Islamabad, Pakistan. Cureus [Internet]. [cited 2025 Apr 27];15(10):e46769.

43. Jordans CCE, Vliegenthart-Jongbloed KJ, van Bruggen AW, van Holten N, van Beek JEA, Vriesde M, et al. Unmasking Individual and Institutional HIV Stigma in Hospitals: Perspectives of Dutch Healthcare Providers. AIDS Behav [Internet]. 2024 [cited 2025 Apr 27];28(9):3184–95.

44. Krishnaratne S, Bond V, Stangl A, Pliakas T, Mathema H, Lilleston P, et al. Stigma and Judgment Toward People Living with HIV and Key Population Groups Among Three Cadres of Health Workers in South Africa and Zambia: Analysis of Data from the HPTN 071 (PopART) Trial. AIDS Patient Care STDs [Internet]. 2020 Jan 1 [cited 2025 May 3];34(1):38–50.

45. Suantari D. Misconceptions and stigma against people living with HIV/AIDS: a cross-sectional study from the 2017 Indonesia Demographic and Health Survey. Epidemiol Health [Internet]. 2021 Nov 6 [cited 2025 May 3];43:e2021094.

46. Tsai AC, Venkataramani AS. The causal effect of education on HIV stigma in Uganda: evidence from a natural experiment. Soc Sci Med 1982 [Internet]. 2015 Oct [cited 2025 May 3];142:37–46.

47. Ebrahimi H, Shoorideh FA, Sohrabi MR, Ebrahimi M, Hosseini M. An Analysis of Approaches to Reduction of HIV Stigma across the World through educational interventions: A Scoping Review. Investig Educ En Enfermeria [Internet]. 2024 Apr 27 [cited 2025 Apr 28];42(1):e06.

48. Srinivasan K, Heylen E, Raj T, Nyblade L, Devadass D, Pereira M, et al. Reduction in stigma drivers partially mediates the effect of a stigma reduction intervention among nursing students in India: The DriSti cluster randomized controlled trial. J Acquir Immune Defic Syndr 1999 [Internet]. 2021 Feb 1 [cited 2025 Apr 28];86(2):182–90.

49. Berg E, Lepp M. The meaning and application of student-centered learning in nursing education: An integrative review of the literature. Nurse Educ Pract [Internet]. 2023 May 1 [cited 2025 Apr 6];69:103622.

50. Yousaf A, Moin H, Majeed S, Shafi R, Mansoor S. The positive impact of introducing modified directed self-learning using pre–small group discussion worksheets as an active learning strategy in undergraduate medical education. Med Educ Online [Internet]. [cited 2025 Apr 29];28(1):2204547.

51. Hammami O, Zaouche K, Kallel M, Nouira M. Évaluation des capacités d'apprentissage après la méthode du jeu de rôle: Comparaison des résultats de l'activité active et de l'observation. Tunis Médicale [Internet]. 2024 Nov [cited 2025 Apr 29];102(11):866–70.

52. Mlouki I, Ayed SB, Chebbi F, Rezg N, Khouildi A, Sassi AH, et al. Hand hygiene and biomedical waste management among medical students: a quasi-experimental study evaluating two training methods. BMC Med Educ [Internet]. 2023 Sep 4 [cited 2025 Jul 27];23:634.

53. Ali MHM, Osman OB, Ibrahim MAEM, Ahmed WAM. The Effect of AIDS Peer Health Education on Knowledge, Attitudes, and Practices of Secondary School Students in Khartoum, Sudan. AIMS Public Health [Internet]. 2015 Oct 22 [cited 2025 Apr 27];2(4):718–26.

54. Batey DS, Whitfield S, Mulla M, Stringer KL, Durojaiye M, McCormick L, et al. Adaptation and Implementation of an Intervention to Reduce HIV-Related Stigma Among Healthcare Workers in the United States: Piloting of the FRESH Workshop. AIDS Patient Care STDs [Internet]. 2016 Nov 1 [cited 2025 Apr 5];30(11):519–27.

55. Doroudi F, Ashourizadeh H, Moosapour S, Ganjizadeh A, Ranjbar-Shourabi A, Parnia A, et al. Gamified Educational Learning Path on HIV/AIDS Stigma and Discrimination (REDXIR): Design, Development and Pilot Study. Med J Islam Repub Iran [Internet]. 2023 Dec 16 [cited 2025 May 1];37:136.

56. Machowska A, Bamboria BL, Bercan C, Sharma M. Impact of 'HIV-related stigma-reduction workshops' on knowledge and attitude of healthcare providers and students in Central India: a pre-test and post-test intervention study. BMJ Open [Internet]. 2020 Apr 1 [cited 2025 Jan 24];10(4):e033612.

57. Platten M, Pham HN, Nguyen HV. Knowledge of HIV and factors associated with attitudes towards HIV among final-year medical students at Hanoi medical university in Vietnam. BMC Public Health [Internet]. 2014 Mar 20 [cited 2025 May 10];14:265.

Supporting Information:

S1 Data. Anonymized Excel dataset (.scv). This file contains the data used to conduct the analyses presented in this study.

Response to reviewers

Stigma against People Living with Human Immunodeficiency Virus: A quasi-experimental evaluation of Active Stigma Reduction Workshops among Medical Students in Tunisia

Dear Dr. Adekola and Reviewers,

We sincerely thank you for your thoughtful and constructive comments. We appreciate the time and expertise invested in reviewing our manuscript. We have carefully revised the manuscript to address all concerns raised. Below, we provide a detailed, point-by-point response. All changes have been highlighted in the revised manuscript with track changes.

Emna Hariz

Response to Editorial Requirements

Formatting and File Naming

The manuscript has been carefully revised to comply fully with PLOS ONE formatting guidelines, including title page structure, section organization, reference formatting, and file naming conventions.

Supporting Information Captions

Captions for all Supporting Information files have been added at the end of the manuscript.

Supporting Information File (S1_Data.csv)

The Supporting Information file S1 has been re-saved. An Excel version has been uploaded.

Reference List Review

The reference list has been thoroughly reviewed for completeness and accuracy.

Response to Academic Editor

Two assumptions the author seem to make, and should revisit:

Comment 1: That statistical significance alone adequately demonstrates intervention value.

We thank the editor for this comment. We have carefully considered this point and addressed it in several places in the manuscript, following suggestions made by the reviewers, to ensure that we discuss both the statistical and practical significance of the intervention.

Comment 2: That readers will intuitively understand the local educational and cultural context.

We agree with the editor's observation and have already made revisions in the methods section to provide clearer explanations of the local educational and cultural context. (page 5, line184).

Response to reviewers:

Reviewer #1:

The manuscript is technically sound, methods well explained and statistical analysis conducted satisfactorily. The results presented support the conclusion as well.

Thank you for your positive feedback.

The manuscript needs few minor revisions, including grammar and spelling checks. There are a few revisions necessary, as follows:

1. Please recheck the grammar and spelling throughout the manuscript.

We have carefully revised the manuscript to correct grammar.

2. In the Method section, please elaborate on the weekly workshop about HIV knowledge and practices- the trainer/teacher qualification, total duration of teaching (in weeks/ months), etc.

We thank the reviewer for this helpful suggestion. We have expanded the Methods section, including the trainer's qualifications (professor of medicine) and the total duration of teaching in one academic year (10 months) (page 3, lines 115 and 123).

3. Page 19, reference 7:- Please add author/organization/website name.

The reference has been revised to include the authoring organization and website name.

4. Please ensure the references are complete and in a standard format.

We have carefully reviewed and standardized all references to ensure completeness and accuracy.

Reviewer #2:

This manuscript addresses an important and underexplored issue: HIV-related stigma among medical students in Tunisia. The focus on the MENA region is timely, and the use of an active, student-centred workshop intervention is appropriate. The quasi-experimental pre–post design and use of validated instruments (HIV-KQ-18 and HPASS) are clear strengths. Overall, the study is sound, but several clarifications are needed to ensure proper interpretation.

We sincerely thank the reviewer for the positive evaluation of our work and for recognizing the relevance and methodological strengths of the study. We appreciate the constructive feedback provided.

First, baseline HIV knowledge is not clearly reported. The statement that 88.4% of participants had "good" knowledge is ambiguous, as it is unclear whether this reflects baseline or post-intervention scores. This must be clarified in the Methods and Results.

We thank the reviewer for highlighting this ambiguity. The 88.4% refers to baseline (pre-intervention) HIV knowledge. This has now been explicitly clarified in the revised manuscript (page 8, line 218).

A 7.9-point reduction in stigma scores (Table 5) is reported as statistically significant, but its practical or clinical relevance is not discussed. Readers need context regarding what constitutes a meaningful change on the HPASS scale.

Action: Discuss the practical significance of the observed stigma reduction and, where possible, compare it with findings from similar studies.

We thank the reviewer for this important comment. We have revised the Discussion section to address the practical significance of the 7.9-point reduction in HPASS scores. We have also contextualized our findings by comparing them with similar interventions in Sudan, the United States, China, and India (page 16, line 385).

All participants were recruited from a single medical faculty. While acknowledged, this limitation is not sufficiently discussed in relation to cultural, institutional, and educational context.

Action: Expand the discussion on how context-specific factors may limit generalizability beyond the Faculty of Medicine of Monastir.

We thank the reviewer for this observation. We have expanded the Discussion section to further elaborate on how sociocultural norms, religious influences, institutional culture, and variations in medical curricula may limit the generalizability of our findings beyond the Faculty of Medicine of Monastir (page 17, lines 418).

The study assesses stigma immediately post-intervention only. Without follow-up, the durability of attitude change cannot be evaluated.

Action: Explicitly acknowledge this limitation and outline plans for follow-up assessments in future studies.

We thank the reviewer for this comment. We have strengthened the Discussion section to explicitly state that stigma was assessed only immediately post-intervention and that the durability of attitude change cannot be determined. We have also added a statement outlining plans for future longitudinal follow-up assessments to evaluate the sustainability of the intervention effects (page 18, line 438).

The relatively low response rate raises concerns about potential selection bias.

Action: Discuss how respondents may differ from non-respondents and the implications for interpretation.

We thank the reviewer for this important comment. We have expanded the Limitations section to acknowledge that the relatively low response rate (43.2%) may introduce selection bias. (page 18, line 442).

The regression results are inconsistently reported (negative coefficient with a confidence interval crossing zero), making interpretation difficult.

Action: Provide a complete regression table including coefficients, confidence intervals, and standard errors for all predictors.

We thank the reviewer for this observation. We have revised the manuscript to include a complete regression table (Table 4) presenting coefficients, 95% confidence intervals, and standard errors.

Results rely primarily on p-values, which limits interpretability.

Action: Add effect size indicators or percentage changes (e.g., relative reduction in stigma scores).

We thank the reviewer for this suggestion. We have added the relative percentage changes in knowledge and stigma scores to the Results section to provide additional context regarding the magnitude of the observed effects (Table 6).

Female students reportedly showed higher stigma levels than males, but no explanation is provided.

Action: Discuss possible cultural or contextual mechanisms underlying this finding.

We thank the reviewer for this insightful comment. We added a discussion of potential sociocultural influences, including gender-related norms surrounding sexuality and HIV in certain contexts, which may shape attitudes. (page 14, line 315).

The very low STI screening rate (2.3%) and heavy reliance on unreliable sources for sexual health information have important implications.

Action: Expand discussion on how these findings inform the need for broader sexual health education.

We thank the reviewer for this comment. We have expanded the Discussion section to highlight that, in our cultural context, sexual health remains a sensitive topic, which may explain the very low STI screening rates and reliance on informal or unreliable sources. We emphasize that these findings underscore the urgent need for broader, culturally appropriate sexual health education among medical students (page 13, line 295).

Comparisons with other settings (e.g., Hong Kong) lack sufficient contextual explanation.

Action: Clarify institutional, cultural, or legal barriers limiting HIV-related clinical exposure in Tunisia.

We thank the reviewer for this comment. We have revised the Discussion section to clarify that institutional, cultural, and legal factors in Tunisia may limit medical students' clinical exposure to HIV patients (page 13, line 274)

There is an apparent discrepancy between the ethics approval date (February 2024) and the reported start of recruitment (September 2023).

Action: Clarify the ethics approval timeline.

We thank the reviewer for highlighting this important point and appreciate the opportunity to clarify. The study protocol was submitted to the institutional ethics committee in 2023, and we received oral approval to proceed while the formal documentation was pending due to procedural delays within the committee. Recruitment only began once we had received this agreement in principle, ensuring that ethical considerations were respected. The official written ethics approval was subsequently issued in February 2024.

The current statement does not clearly meet PLOS ONE requirements for public data access.

Action: Specify the public repository and confirm unrestricted access.

We thank the reviewer for this comment. The Data Availability Statement has been revised to clearly indicate that all underlying data are fully available in the Supporting Information file, including the saved Excel dataset (S1_Data.csv), ensuring unrestricted access (page 19, line 458).

Reviewer #3:

This manuscript is well written. The abstract was a representative summary of the manuscript. The background provided sufficient clarity on the rationale and objective of the study. The

methods were adequately described to enable reproducibility. The findings were well discoursed and situated in existing literature.

Your constructive appraisal is highly valued and motivates us to continue improving the quality and rigor of our work.
