# Supplementary material for: Stigma against people living with Human Immunodeficiency Virus: A quasi-experimental evaluation of active stigma reduction workshops among medical students in Tunisia
Source: PLoS One. 2026 Jun 11;21(6):e0350810. doi: 10.1371/journal.pone.0350810 (PMC13257959; doi:10.1371/journal.pone.0350810)
Supplement: Attachment — Submitted filename: Reviewer comments [PONE-D-25-49448].docx [file pone.0350810.s003.docx]

**Reviewer Comments**

**Manuscript ID:** PONE-D-25-49448
**Recommendation:** *Minor Revision*

**General Assessment**

This manuscript addresses an important and underexplored issue: HIV-related stigma among medical students in Tunisia. The focus on the MENA region is timely, and the use of an active, student-centred workshop intervention is appropriate. The quasi-experimental pre–post design and use of validated instruments (HIV-KQ-18, HPASS) are notable strengths.

However, several issues related to clarity, interpretation, and reporting need to be addressed before the manuscript can be considered for publication.

**Major Comments**

1. **Baseline HIV knowledge not clearly reported**
   The manuscript reports that 88.4% of participants had “good” HIV knowledge, but it is unclear whether this refers to baseline or post-intervention scores. This information is essential to assess the intervention’s impact.

**Action:** The authors must clearly report baseline HIV knowledge scores in the Methods and Results sections.

2. **Interpretation of stigma reduction**
   A 7.9-point reduction in stigma scores (Table 5) is reported as statistically significant, but its practical or clinical relevance is not discussed. Readers need context regarding what constitutes a meaningful change on the HPASS scale.

**Action:** Discuss the practical significance of the observed stigma reduction and, where possible, compare it with findings from similar studies.

3. **Limited generalizability due to single-centre design**
   All participants were recruited from a single medical faculty. While acknowledged, this limitation is not sufficiently discussed in relation to cultural, institutional, and educational context.

**Action:** Expand the discussion on how context-specific factors may limit generalizability beyond the Faculty of Medicine of Monastir.

4. **No assessment of sustained impact**
   The study assesses stigma immediately post-intervention only. Without follow-up, the durability of attitude change cannot be evaluated.

**Action:** Explicitly acknowledge this limitation and outline plans for follow-up assessments in future studies.

**Methods and Reporting Issues**

5. **Low response rate (43.2%)**
   The relatively low response rate raises concerns about potential selection bias.

**Action:** Discuss how respondents may differ from non-respondents and the implications for interpretation.

6. **Regression analysis reporting unclear**
   The regression results are inconsistently reported (negative coefficient with a confidence interval crossing zero), making interpretation difficult.

**Action:** Provide a complete regression table including coefficients, confidence intervals, and standard errors for all predictors.

7. **Effect size presentation**
   Results rely primarily on p-values, which limits interpretability.

**Action:** Add effect size indicators or percentage changes (e.g., relative reduction in stigma scores).

**Interpretation and Context**

8. **Gender differences**
   Female students reportedly showed higher stigma levels than males, but no explanation is provided.

**Action:** Discuss possible cultural or contextual mechanisms underlying this finding.

9. **Low STI screening and information sources**
   The very low STI screening rate (2.3%) and heavy reliance on unreliable sources for sexual health information have important implications.

**Action:** Expand discussion on how these findings inform the need for broader sexual health education.

10. **Cultural barriers to HIV exposure**
    Comparisons with other settings (e.g., Hong Kong) lack sufficient contextual explanation.

**Action:** Clarify institutional, cultural, or legal barriers limiting HIV-related clinical exposure in Tunisia.

**Ethics and Data Availability**

11. **Ethics approval timeline**
    There is an apparent discrepancy between the ethics approval date (February 2024) and the reported start of recruitment (September 2023).

**Action:** Clarify the ethics approval timeline.

12. **Data availability statement**
    The current statement does not clearly meet PLOS ONE requirements for public data access.

**Action:** Specify the public repository and confirm unrestricted access.

**Summary**

This study contributes valuable evidence on HIV stigma reduction among medical students in the MENA region. With improved clarity on baseline measures, stronger contextual interpretation, and corrections to statistical and ethics reporting, the manuscript would be suitable for publication.
