# Supplementary material for: Stigma against people living with Human Immunodeficiency Virus: A quasi-experimental evaluation of active stigma reduction workshops among medical students in Tunisia
Source: PLoS One. 2026 Jun 11;21(6):e0350810. doi: 10.1371/journal.pone.0350810 (PMC13257959; doi:10.1371/journal.pone.0350810)
Supplement: Attachment — Submitted filename: Reviewer Comments D-25 49448- JLA.docx [file pone.0350810.s004.docx]

The manuscript covers an essential topic and the authors have made a good effort to study it.

There are a few revisions necessary, as follows:

1. Please recheck the grammar and spelling throughout the manuscript.
2. In the Method section, please elaborate on the weekly workshop about HIV knowledge and practices- the trainer/teacher qualification, total duration of teaching (in weeks/ months), etc.
3. Page 19, reference 7:- Please add author/organization/website name.
4. Please ensure the references are complete and in a standard format.
