# Supplementary material for: Stigma against people living with Human Immunodeficiency Virus: A quasi-experimental evaluation of active stigma reduction workshops among medical students in Tunisia
Source: PLoS One. 2026 Jun 11;21(6):e0350810. doi: 10.1371/journal.pone.0350810 (PMC13257959; doi:10.1371/journal.pone.0350810)
Supplement: Attachment — Submitted filename: Response to reviewers.docx [file pone.0350810.s005.docx]

**Response to reviewers**

**Stigma against People Living with Human Immunodeficiency Virus: A quasi-experimental evaluation of Active Stigma Reduction Workshops among Medical Students in Tunisia**

Dear Dr. Adekola and Reviewers,
We sincerely thank you for your thoughtful and constructive comments. We appreciate the time and expertise invested in reviewing our manuscript. We have carefully revised the manuscript to address all concerns raised. Below, we provide a detailed, point-by-point response. All changes have been highlighted in the revised manuscript with track changes.

Emna Hariz

**Response to Editorial Requirements**

Formatting and File Naming

The manuscript has been carefully revised to comply fully with PLOS ONE formatting guidelines, including title page structure, section organization, reference formatting, and file naming conventions.

Supporting Information Captions

Captions for all Supporting Information files have been added at the end of the manuscript.

Supporting Information File (S1_Data.csv)

The Supporting Information file S1 has been re-saved. An Excel version has been uploaded.

Reference List Review

The reference list has been thoroughly reviewed for completeness and accuracy.

**Response to Academic Editor**

Two assumptions the author seem to make, and should revisit:

Comment 1: That statistical significance alone adequately demonstrates intervention value.

We thank the editor for this comment. We have carefully considered this point and addressed it in several places in the manuscript, following suggestions made by the reviewers, to ensure that we discuss both the statistical and practical significance of the intervention.

Comment 2: That readers will intuitively understand the local educational and cultural context.

We agree with the editor’s observation and have already made revisions in the methods section to provide clearer explanations of the local educational and cultural context. (page 5, line184).

**Response to reviewers:**

**Reviewer #1:**

The manuscript is technically sound, methods well explained and statistical analysis conducted satisfactorily. The results presented support the conclusion as well.

Thank you for your positive feedback.

The manuscript needs few minor revisions, including grammar and spelling checks. There are a few revisions necessary, as follows:

1. Please recheck the grammar and spelling throughout the manuscript.

We have carefully revised the manuscript to correct grammar.

2. In the Method section, please elaborate on the weekly workshop about HIV knowledge and practices- the trainer/teacher qualification, total duration of teaching (in weeks/ months), etc.

We thank the reviewer for this helpful suggestion. We have expanded the Methods section, including the trainer’s qualifications (professor of medicine) and the total duration of teaching in one academic year (10 months) (page 3, lines 115 and 123).

3. Page 19, reference 7:- Please add author/organization/website name.

The reference has been revised to include the authoring organization and website name.

4. Please ensure the references are complete and in a standard format.

We have carefully reviewed and standardized all references to ensure completeness and accuracy.

**Reviewer #2:**

This manuscript addresses an important and underexplored issue: HIV-related stigma among medical students in Tunisia. The focus on the MENA region is timely, and the use of an active, student-centred workshop intervention is appropriate. The quasi-experimental pre–post design and use of validated instruments (HIV-KQ-18 and HPASS) are clear strengths. Overall, the study is sound, but several clarifications are needed to ensure proper interpretation.

We sincerely thank the reviewer for the positive evaluation of our work and for recognizing the relevance and methodological strengths of the study. We appreciate the constructive feedback provided.

First, baseline HIV knowledge is not clearly reported. The statement that 88.4% of participants had “good” knowledge is ambiguous, as it is unclear whether this reflects baseline or post-intervention scores. This must be clarified in the Methods and Results.

We thank the reviewer for highlighting this ambiguity. The 88.4% refers to baseline (pre-intervention) HIV knowledge. This has now been explicitly clarified in the revised manuscript (page 8, line 218).

A 7.9-point reduction in stigma scores (Table 5) is reported as statistically significant, but its practical or clinical relevance is not discussed. Readers need context regarding what constitutes a meaningful change on the HPASS scale.

**Action:** Discuss the practical significance of the observed stigma reduction and, where possible, compare it with findings from similar studies.

We thank the reviewer for this important comment. We have revised the Discussion section to address the practical significance of the 7.9-point reduction in HPASS scores. We have also contextualized our findings by comparing them with similar interventions in Sudan, the United States, China, and India (page 16, line 385).

All participants were recruited from a single medical faculty. While acknowledged, this limitation is not sufficiently discussed in relation to cultural, institutional, and educational context.

**Action:** Expand the discussion on how context-specific factors may limit generalizability beyond the Faculty of Medicine of Monastir.

We thank the reviewer for this observation. We have expanded the Discussion section to further elaborate on how sociocultural norms, religious influences, institutional culture, and variations in medical curricula may limit the generalizability of our findings beyond the Faculty of Medicine of Monastir (page 17, lines 418).

The study assesses stigma immediately post-intervention only. Without follow-up, the durability of attitude change cannot be evaluated.

**Action:** Explicitly acknowledge this limitation and outline plans for follow-up assessments in future studies.

We thank the reviewer for this comment. We have strengthened the Discussion section to explicitly state that stigma was assessed only immediately post-intervention and that the durability of attitude change cannot be determined. We have also added a statement outlining plans for future longitudinal follow-up assessments to evaluate the sustainability of the intervention effects (page 18, line 438).

The relatively low response rate raises concerns about potential selection bias.

**Action:** Discuss how respondents may differ from non-respondents and the implications for interpretation.

We thank the reviewer for this important comment. We have expanded the Limitations section to acknowledge that the relatively low response rate (43.2%) may introduce selection bias. (page 18, line 442).

The regression results are inconsistently reported (negative coefficient with a confidence interval crossing zero), making interpretation difficult.

**Action:** Provide a complete regression table including coefficients, confidence intervals, and standard errors for all predictors.

We thank the reviewer for this observation. We have revised the manuscript to include a complete regression table (Table 4) presenting coefficients, 95% confidence intervals, and standard errors.

Results rely primarily on p-values, which limits interpretability.

**Action:** Add effect size indicators or percentage changes (e.g., relative reduction in stigma scores).

We thank the reviewer for this suggestion. We have added the relative percentage changes in knowledge and stigma scores to the Results section to provide additional context regarding the magnitude of the observed effects (Table 6).

Female students reportedly showed higher stigma levels than males, but no explanation is provided.

**Action:** Discuss possible cultural or contextual mechanisms underlying this finding.

We thank the reviewer for this insightful comment. We added a discussion of potential sociocultural influences, including gender-related norms surrounding sexuality and HIV in certain contexts, which may shape attitudes. (page 14, line 315).

The very low STI screening rate (2.3%) and heavy reliance on unreliable sources for sexual health information have important implications.

**Action:** Expand discussion on how these findings inform the need for broader sexual health education.

We thank the reviewer for this comment. We have expanded the Discussion section to highlight that, in our cultural context, sexual health remains a sensitive topic, which may explain the very low STI screening rates and reliance on informal or unreliable sources. We emphasize that these findings underscore the urgent need for broader, culturally appropriate sexual health education among medical students (page 13, line 295).

Comparisons with other settings (e.g., Hong Kong) lack sufficient contextual explanation.

**Action:** Clarify institutional, cultural, or legal barriers limiting HIV-related clinical exposure in Tunisia.

We thank the reviewer for this comment. We have revised the Discussion section to clarify that institutional, cultural, and legal factors in Tunisia may limit medical students’ clinical exposure to HIV patients (page 13, line 274)

There is an apparent discrepancy between the ethics approval date (February 2024) and the reported start of recruitment (September 2023).

**Action:** Clarify the ethics approval timeline.

We thank the reviewer for highlighting this important point and appreciate the opportunity to clarify. The study protocol was submitted to the institutional ethics committee in 2023, and we received oral approval to proceed while the formal documentation was pending due to procedural delays within the committee. Recruitment only began once we had received this agreement in principle, ensuring that ethical considerations were respected. The official written ethics approval was subsequently issued in February 2024.

The current statement does not clearly meet PLOS ONE requirements for public data access.

**Action:** Specify the public repository and confirm unrestricted access.

We thank the reviewer for this comment. The Data Availability Statement has been revised to clearly indicate that all underlying data are fully available in the Supporting Information file, including the saved Excel dataset (S1_Data.csv), ensuring unrestricted access (page 19, line 458).

**Reviewer #3:**

This manuscript is well written. The abstract was a representative summary of the manuscript. The background provided sufficient clarity on the rationale and objective of the study. The methods were adequately described to enable reproducibility. The findings were well discoursed and situated in existing literature.

Your constructive appraisal is highly valued and motivates us to continue improving the quality and rigor of our work.
