# Supplementary material for: Stigma against people living with Human Immunodeficiency Virus: A quasi-experimental evaluation of active stigma reduction workshops among medical students in Tunisia
Source: PLoS One. 2026 Jun 11;21(6):e0350810. doi: 10.1371/journal.pone.0350810 (PMC13257959; doi:10.1371/journal.pone.0350810)
Supplement: Peer review history

# Peer review: Decision Letter 0

**DOI:** 10.1371/journal.pone.0350810.r001

**Authors:** Ayobami Precious Adekola

27 Jan 2026

PONE-D-25-49448

Stigma against People Living with Human Immunodeficiency Virus: A quasi-experimental evaluation of Active Stigma Reduction Workshops among Medical Students in Tunisia

PLOS One

Dear Dr. Hariz,

Thank you for submitting your manuscript to PLOS ONE. After careful consideration, we feel that it has merit but does not fully meet PLOS ONE’s publication criteria as it currently stands. Therefore, we invite you to submit a revised version of the manuscript that addresses the points raised during the review process.

==============================

Two assumptions the author seem to make, and should revisit:

1. That statistical significance alone adequately demonstrates intervention value.
2. That readers will intuitively understand the local educational and cultural context.

Neither assumption holds for an international readership.

==============================

Please submit your revised manuscript by Mar 13 2026 11:59PM. If you will need more time than this to complete your revisions, please reply to this message or contact the journal office at plosone@plos.org. When you're ready to submit your revision, log on to https://www.editorialmanager.com/pone/ and select the 'Submissions Needing Revision' folder to locate your manuscript file.

Please include the following items when submitting your revised manuscript:

- A letter that responds to each point raised by the academic editor and reviewer(s). You should upload this letter as a separate file labeled 'Response to Reviewers'.
- A marked-up copy of your manuscript that highlights changes made to the original version. You should upload this as a separate file labeled 'Revised Manuscript with Track Changes'.
- An unmarked version of your revised paper without tracked changes. You should upload this as a separate file labeled 'Manuscript'.

If you would like to make changes to your financial disclosure, please include your updated statement in your cover letter. Guidelines for resubmitting your figure files are available below the reviewer comments at the end of this letter.

-->If applicable, we recommend that you deposit your laboratory protocols in protocols.io to enhance the reproducibility of your results. Protocols.io assigns your protocol its own identifier (DOI) so that it can be cited independently in the future. For instructions see: https://journals.plos.org/plosone/s/submission-guidelines#loc-laboratory-protocols. Additionally, PLOS ONE offers an option for publishing peer-reviewed Lab Protocol articles, which describe protocols hosted on protocols.io. Read more information on sharing protocols at https://plos.org/protocols?utm_medium=editorial-email&utm_source=authorletters&utm_campaign=protocols.

We look forward to receiving your revised manuscript.

Kind regards,

Ayobami Precious Adekola, PhD in Public Health

Academic Editor

PLOS One

Journal Requirements:

When submitting your revision, we need you to address these additional requirements.

1. Please ensure that your manuscript meets PLOS ONE's style requirements, including those for file naming. The PLOS ONE style templates can be found at

https://journals.plos.org/plosone/s/file?id=wjVg/PLOSOne_formatting_sample_main_body.pdf and

https://journals.plos.org/plosone/s/file?id=ba62/PLOSOne_formatting_sample_title_authors_affiliations.pdf

2. Please include captions for your Supporting Information files at the end of your manuscript, and update any in-text citations to match accordingly. Please see our Supporting Information guidelines for more information: http://journals.plos.org/plosone/s/supporting-information.

3. We are unable to open your Supporting Information file S1_file.sav. Please kindly revise as necessary and re-upload.

4. If the reviewer comments include a recommendation to cite specific previously published works, please review and evaluate these publications to determine whether they are relevant and should be cited. There is no requirement to cite these works unless the editor has indicated otherwise.

5. Please review your reference list to ensure that it is complete and correct. If you have cited papers that have been retracted, please include the rationale for doing so in the manuscript text, or remove these references and replace them with relevant current references. Any changes to the reference list should be mentioned in the rebuttal letter that accompanies your revised manuscript. If you need to cite a retracted article, indicate the article’s retracted status in the References list and also include a citation and full reference for the retraction notice.

Additional Editor Comments:

Dear Author,

Thank you for submitting your manuscript to PLOS ONE. Following peer review, I have recommended that your manuscript be considered for publication provided that all issues raised by the reviewers are adequately addressed.

Please ensure that your revision responds comprehensively to each comment in the reviewers’ reports.

We look forward to receiving your revised manuscript.

Kind regards,

Dr Ayobami Adekola

[Note: HTML markup is below. Please do not edit.]

Reviewers' comments:

Reviewer's Responses to Questions

**Comments to the Author**

1. Is the manuscript technically sound, and do the data support the conclusions?

The manuscript must describe a technically sound piece of scientific research with data that supports the conclusions. Experiments must have been conducted rigorously, with appropriate controls, replication, and sample sizes. The conclusions must be drawn appropriately based on the data presented.

Reviewer #1: Yes

Reviewer #2: Yes

Reviewer #3: Yes

**********

2. Has the statistical analysis been performed appropriately and rigorously?

Reviewer #1: Yes

Reviewer #2: Yes

Reviewer #3: Yes

**********

3. Have the authors made all data underlying the findings in their manuscript fully available?

The PLOS Data policy requires authors to make all data underlying the findings described in their manuscript fully available without restriction, with rare exception (please refer to the Data Availability Statement in the manuscript PDF file). The data should be provided as part of the manuscript or its supporting information, or deposited to a public repository. For example, in addition to summary statistics, the data points behind means, medians and variance measures should be available. If there are restrictions on publicly sharing data—e.g. participant privacy or use of data from a third party—those must be specified.

Reviewer #1: Yes

Reviewer #2: Yes

Reviewer #3: Yes

**********

4. Is the manuscript presented in an intelligible fashion and written in standard English?

PLOS ONE does not copyedit accepted manuscripts, so the language in submitted articles must be clear, correct, and unambiguous. Any typographical or grammatical errors should be corrected at revision, so please note any specific errors here.

Reviewer #1: Yes

Reviewer #2: Yes

Reviewer #3: Yes

**********

5. Review Comments to the Author

Please use the space provided to explain your answers to the questions above. You may also include additional comments for the author, including concerns about dual publication, research ethics, or publication ethics. (Please upload your review as an attachment if it exceeds 20,000 characters)

Reviewer #1: The manuscript is technically sound, methods well explained and statistical analysis conducted satisfactorily. The results presented support the conclusion as well. The manuscript needs few minor revisions, including grammar and spelling checks. The

Reviewer #2: This manuscript addresses an important and underexplored issue: HIV-related stigma among medical students in Tunisia. The focus on the MENA region is timely, and the use of an active, student-centred workshop intervention is appropriate. The quasi-experimental pre–post design and use of validated instruments (HIV-KQ-18 and HPASS) are clear strengths. Overall, the study is sound, but several clarifications are needed to ensure proper interpretation.

First, baseline HIV knowledge is not clearly reported. The statement that 88.4% of participants had “good” knowledge is ambiguous, as it is unclear whether this reflects baseline or post-intervention scores. This must be clarified in the Methods and Results.

Second, while a statistically significant 7.9-point reduction in stigma scores is reported, the practical significance of this change is not discussed. The authors should explain what constitutes a meaningful change on the HPASS scale and, where possible, relate this to findings from similar studies.

Third, the single-centre design limits generalizability. All participants were drawn from one medical faculty, and contextual factors such as institutional culture, religious norms, and exposure to HIV care may strongly influence stigma levels. This limitation should be discussed more explicitly.

Fourth, the absence of follow-up data prevents conclusions about the sustainability of stigma reduction or behavioural change. This should be acknowledged, with future follow-up plans outlined.

Additional concerns include the low response rate (43.2%), suggesting potential selection bias; unclear regression reporting requiring a complete regression table; and reliance on p-values without clear effect size interpretation. The reported gender difference in stigma and the very low STI screening rate also warrant discussion.

Finally, the ethics approval timeline and data availability statement need clarification to ensure compliance with PLOS ONE requirements.

With these revisions, the manuscript would make a valuable contribution to HIV stigma reduction research in medical education.

Reviewer #3: This manuscript is well written. The abstract was a representative summary of the manuscript. The background provided sufficient clarity on the rationale and objective of the study. The methods were adequately described to enable reproducibility. The findings were well discoursed and situated in existing literature.

**********

6. PLOS authors have the option to publish the peer review history of their article (what does this mean?). If published, this will include your full peer review and any attached files.

If you choose “no”, your identity will remain anonymous but your review may still be made public.

**Do you want your identity to be public for this peer review?** For information about this choice, including consent withdrawal, please see our Privacy Policy.

Reviewer #1: No

Reviewer #2: **Yes:** ABENA FOE Jean-Louis

Reviewer #3: **Yes:** Oluwasanmi Akanji Adedokun

**********

[NOTE: If reviewer comments were submitted as an attachment file, they will be attached to this email and accessible via the submission site. Please log into your account, locate the manuscript record, and check for the action link "View Attachments". If this link does not appear, there are no attachment files.]

To ensure your figures meet our technical requirements, please review our figure guidelines: https://journals.plos.org/plosone/s/figures

You may also use PLOS’s free figure tool, NAAS, to help you prepare publication quality figures: https://journals.plos.org/plosone/s/figures#loc-tools-for-figure-preparation.

NAAS will assess whether your figures meet our technical requirements by comparing each figure against our figure specifications.

Attachment Submitted filename: Reviewer comments [PONE-D-25-49448].docx

Attachment Submitted filename: Reviewer Comments D-25 49448- JLA.docx

# Author comment: Author response to Decision Letter 1

**DOI:** 10.1371/journal.pone.0350810.r002

23 Mar 2026

Dear Dr. Adekola and Reviewers,

We sincerely thank you for your thoughtful and constructive comments. We appreciate the time and expertise invested in reviewing our manuscript. We have carefully revised the manuscript to address all concerns raised. Below, we provide a detailed, point-by-point response. All changes have been highlighted in the revised manuscript with track changes.

Emna Hariz

Response to Editorial Requirements

Formatting and File Naming

The manuscript has been carefully revised to comply fully with PLOS ONE formatting guidelines, including title page structure, section organization, reference formatting, and file naming conventions.

Supporting Information Captions

Captions for all Supporting Information files have been added at the end of the manuscript.

Supporting Information File (S1_Data.csv)

The Supporting Information file S1 has been re-saved. An Excel version has been uploaded.

Reference List Review

The reference list has been thoroughly reviewed for completeness and accuracy.

Response to Academic Editor

Two assumptions the author seem to make, and should revisit:

Comment 1: That statistical significance alone adequately demonstrates intervention value.

We thank the editor for this comment. We have carefully considered this point and addressed it in several places in the manuscript, following suggestions made by the reviewers, to ensure that we discuss both the statistical and practical significance of the intervention.

Comment 2: That readers will intuitively understand the local educational and cultural context.

We agree with the editor’s observation and have already made revisions in the methods section to provide clearer explanations of the local educational and cultural context. (page 5, line184).

Response to reviewers:

Reviewer #1:

The manuscript is technically sound, methods well explained and statistical analysis conducted satisfactorily. The results presented support the conclusion as well.

Thank you for your positive feedback.

The manuscript needs few minor revisions, including grammar and spelling checks. There are a few revisions necessary, as follows:

1. Please recheck the grammar and spelling throughout the manuscript.

We have carefully revised the manuscript to correct grammar.

2. In the Method section, please elaborate on the weekly workshop about HIV knowledge and practices- the trainer/teacher qualification, total duration of teaching (in weeks/ months), etc.

We thank the reviewer for this helpful suggestion. We have expanded the Methods section, including the trainer’s qualifications (professor of medicine) and the total duration of teaching in one academic year (10 months) (page 3, lines 115 and 123).

3. Page 19, reference 7:- Please add author/organization/website name.

The reference has been revised to include the authoring organization and website name.

4. Please ensure the references are complete and in a standard format.

We have carefully reviewed and standardized all references to ensure completeness and accuracy.

Reviewer #2:

This manuscript addresses an important and underexplored issue: HIV-related stigma among medical students in Tunisia. The focus on the MENA region is timely, and the use of an active, student-centred workshop intervention is appropriate. The quasi-experimental pre–post design and use of validated instruments (HIV-KQ-18 and HPASS) are clear strengths. Overall, the study is sound, but several clarifications are needed to ensure proper interpretation.

We sincerely thank the reviewer for the positive evaluation of our work and for recognizing the relevance and methodological strengths of the study. We appreciate the constructive feedback provided.

First, baseline HIV knowledge is not clearly reported. The statement that 88.4% of participants had “good” knowledge is ambiguous, as it is unclear whether this reflects baseline or post-intervention scores. This must be clarified in the Methods and Results.

We thank the reviewer for highlighting this ambiguity. The 88.4% refers to baseline (pre-intervention) HIV knowledge. This has now been explicitly clarified in the revised manuscript (page 8, line 218).

A 7.9-point reduction in stigma scores (Table 5) is reported as statistically significant, but its practical or clinical relevance is not discussed. Readers need context regarding what constitutes a meaningful change on the HPASS scale.

Action: Discuss the practical significance of the observed stigma reduction and, where possible, compare it with findings from similar studies.

We thank the reviewer for this important comment. We have revised the Discussion section to address the practical significance of the 7.9-point reduction in HPASS scores. We have also contextualized our findings by comparing them with similar interventions in Sudan, the United States, China, and India (page 16, line 385).

All participants were recruited from a single medical faculty. While acknowledged, this limitation is not sufficiently discussed in relation to cultural, institutional, and educational context.

Action: Expand the discussion on how context-specific factors may limit generalizability beyond the Faculty of Medicine of Monastir.

We thank the reviewer for this observation. We have expanded the Discussion section to further elaborate on how sociocultural norms, religious influences, institutional culture, and variations in medical curricula may limit the generalizability of our findings beyond the Faculty of Medicine of Monastir (page 17, lines 418).

The study assesses stigma immediately post-intervention only. Without follow-up, the durability of attitude change cannot be evaluated.

Action: Explicitly acknowledge this limitation and outline plans for follow-up assessments in future studies.

We thank the reviewer for this comment. We have strengthened the Discussion section to explicitly state that stigma was assessed only immediately post-intervention and that the durability of attitude change cannot be determined. We have also added a statement outlining plans for future longitudinal follow-up assessments to evaluate the sustainability of the intervention effects (page 18, line 438).

The relatively low response rate raises concerns about potential selection bias.

Action: Discuss how respondents may differ from non-respondents and the implications for interpretation.

We thank the reviewer for this important comment. We have expanded the Limitations section to acknowledge that the relatively low response rate (43.2%) may introduce selection bias. (page 18, line 442).

The regression results are inconsistently reported (negative coefficient with a confidence interval crossing zero), making interpretation difficult.

Action: Provide a complete regression table including coefficients, confidence intervals, and standard errors for all predictors.

We thank the reviewer for this observation. We have revised the manuscript to include a complete regression table (Table 4) presenting coefficients, 95% confidence intervals, and standard errors.

Results rely primarily on p-values, which limits interpretability.

Action: Add effect size indicators or percentage changes (e.g., relative reduction in stigma scores).

We thank the reviewer for this suggestion. We have added the relative percentage changes in knowledge and stigma scores to the Results section to provide additional context regarding the magnitude of the observed effects (Table 6).

Female students reportedly showed higher stigma levels than males, but no explanation is provided.

Action: Discuss possible cultural or contextual mechanisms underlying this finding.

We thank the reviewer for this insightful comment. We added a discussion of potential sociocultural influences, including gender-related norms surrounding sexuality and HIV in certain contexts, which may shape attitudes. (page 14, line 315).

The very low STI screening rate (2.3%) and heavy reliance on unreliable sources for sexual health information have important implications.

Action: Expand discussion on how these findings inform the need for broader sexual health education.

We thank the reviewer for this comment. We have expanded the Discussion section to highlight that, in our cultural context, sexual health remains a sensitive topic, which may explain the very low STI screening rates and reliance on informal or unreliable sources. We emphasize that these findings underscore the urgent need for broader, culturally appropriate sexual health education among medical students (page 13, line 295).

Comparisons with other settings (e.g., Hong Kong) lack sufficient contextual explanation.

Action: Clarify institutional, cultural, or legal barriers limiting HIV-related clinical exposure in Tunisia.

We thank the reviewer for this comment. We have revised the Discussion section to clarify that institutional, cultural, and legal factors in Tunisia may limit medical students’ clinical exposure to HIV patients (page 13, line 274)

There is an apparent discrepancy between the ethics approval date (February 2024) and the reported start of recruitment (September 2023).

Action: Clarify the ethics approval timeline.

We thank the reviewer for highlighting this important point and appreciate the opportunity to clarify. The study protocol was submitted to the institutional ethics committee in 2023, and we received oral approval to proceed while the formal documentation was pending due to procedural delays within the committee. Recruitment only began once we had received this agreement in principle, ensuring that ethical considerations were respected. The official written ethics approval was subsequently issued in February 2024.

The current statement does not clearly meet PLOS ONE requirements for public data access.

Action: Specify the public repository and confirm unrestricted access.

We thank the reviewer for this comment. The Data Availability Statement has been revised to clearly indicate that all underlying data are fully available in the Supporting Information file, including the saved Excel dataset (S1_Data.csv), ensuring unrestricted access (page 19, line 458).

Reviewer #3:

This manuscript is well written. The abstract was a representative summary of the manuscript. The background provided sufficient clarity on the rationale and objective of the study. The methods were adequately described to enable reproducibility. The findings were well discoursed and situated in existing literature.

Your constructive appraisal is highly valued and motivates us to continue improving the quality and rigor of our work.

Attachment Submitted filename: Response to reviewers.docx

# Peer review: Decision Letter 1

**DOI:** 10.1371/journal.pone.0350810.r003

**Authors:** Hamufare Dumisani Mugauri

19 May 2026

Stigma against People Living with Human Immunodeficiency Virus: A quasi-experimental evaluation of Active Stigma Reduction Workshops among Medical Students in Tunisia

PONE-D-25-49448R1

Dear Dr. Hariz,

We’re pleased to inform you that your manuscript has been judged scientifically suitable for publication and will be formally accepted for publication once it meets all outstanding technical requirements.

Within one week, you’ll receive an e-mail detailing the required amendments. When these have been addressed, you’ll receive a formal acceptance letter and your manuscript will be scheduled for publication.

An invoice will be generated when your article is formally accepted. Please note, if your institution has a publishing partnership with PLOS and your article meets the relevant criteria, all or part of your publication costs will be covered. Please make sure your user information is up-to-date by logging into Editorial Manager at Editorial Manager® and clicking the ‘Update My Information' link at the top of the page. For questions related to billing, please contact billing support.

If your institution or institutions have a press office, please notify them about your upcoming paper to help maximize its impact. If they’ll be preparing press materials, please inform our press team as soon as possible -- no later than 48 hours after receiving the formal acceptance. Your manuscript will remain under strict press embargo until 2 pm Eastern Time on the date of publication. For more information, please contact onepress@plos.org.

Kind regards,

Hamufare Dumisani Mugauri, Ph.D. Epidemiology and Public Health

Academic Editor

PLOS One

Additional Editor Comments (optional):

Reviewers' comments:

Reviewer's Responses to Questions

**Comments to the Author**

1. If the authors have adequately addressed your comments raised in a previous round of review and you feel that this manuscript is now acceptable for publication, you may indicate that here to bypass the “Comments to the Author” section, enter your conflict of interest statement in the “Confidential to Editor” section, and submit your "Accept" recommendation.

Reviewer #1: All comments have been addressed

Reviewer #2: All comments have been addressed

Reviewer #3: All comments have been addressed

**********

2. Is the manuscript technically sound, and do the data support the conclusions?

The manuscript must describe a technically sound piece of scientific research with data that supports the conclusions. Experiments must have been conducted rigorously, with appropriate controls, replication, and sample sizes. The conclusions must be drawn appropriately based on the data presented.

Reviewer #1: Yes

Reviewer #2: Partly

Reviewer #3: Yes

**********

3. Has the statistical analysis been performed appropriately and rigorously?

Reviewer #1: Yes

Reviewer #2: Yes

Reviewer #3: Yes

**********

4. Have the authors made all data underlying the findings in their manuscript fully available?

The PLOS Data policy requires authors to make all data underlying the findings described in their manuscript fully available without restriction, with rare exception (please refer to the Data Availability Statement in the manuscript PDF file). The data should be provided as part of the manuscript or its supporting information, or deposited to a public repository. For example, in addition to summary statistics, the data points behind means, medians and variance measures should be available. If there are restrictions on publicly sharing data—e.g. participant privacy or use of data from a third party—those must be specified.

Reviewer #1: Yes

Reviewer #2: (No Response)

Reviewer #3: Yes

**********

5. Is the manuscript presented in an intelligible fashion and written in standard English?

PLOS ONE does not copyedit accepted manuscripts, so the language in submitted articles must be clear, correct, and unambiguous. Any typographical or grammatical errors should be corrected at revision, so please note any specific errors here.

Reviewer #1: Yes

Reviewer #2: Yes

Reviewer #3: Yes

**********

6. Review Comments to the Author

Please use the space provided to explain your answers to the questions above. You may also include additional comments for the author, including concerns about dual publication, research ethics, or publication ethics. (Please upload your review as an attachment if it exceeds 20,000 characters)

Reviewer #1: The authors have addressed the review comments. Suitable changes and corrections have been made. Manuscript is acceptable for publication.

Reviewer #2: Review comments

Reviewer Memo to the Handling Editor

Manuscript: PONE-D-25-49448R1 — Stigma against PLHIV: Quasi-experimental evaluation of Active Stigma Reduction Workshops among Medical Students in Tunisia

Reviewer assessment of R1 response coverage

Date: 11 May 2026

Overall judgment

The authors have engaged seriously with the previous round and the manuscript is substantially improved. Most of the substantive concerns from Reviewer #2 and the Academic Editor have been addressed in the text, and the addition of Table 6 (relative changes) and Table 4 (regression with CIs/SE) materially strengthens interpretability. However, several residual issues remain that should be resolved before acceptance, including one internal data inconsistency and one regression reporting anomaly.

Recommendation: Minor Revision.

Coverage scorecard

#CommentCoverageNoteAE-1Statistical vs practical significancePartial7.9-pt reduction framed as "tangible" but no MCID or quantitative anchor for HPASSAE-2Local educational/cultural contextDoneNew paragraph p.5, l.184R1-1Grammar/spellingIncompleteResidual typos: "attitudestowards" (l.305, l.339), "decease" (l.361), ".scv" (l.668)R1-2Trainer qualifications, durationDone"Professor of medicine"; HIV-specific expertise still vagueR1-3Reference 7DoneECDC now citedR1-4References standardisedPartialRef 20 still a ResearchGate link with chatgpt.com utm; Ref 1 same utm artefact; Ref 27 incompleteR2-1Baseline 88.4% clarifiedDonel.218R2-2Practical significance + cross-study comparisonPartialComparators added (Sudan, US, China, India) but no comparable effect-size magnitudesR2-3Single-faculty generalisabilityDoneExpanded l.418R2-4Durability / follow-up plansDonel.438R2-5Low response rate / selection biasPartialOne sentence (l.442); no real discussion of respondent vs non-respondent differencesR2-6Full regression tableProblematicTable 4: B = 0.359 with 95% CI 0.27–21.05 — scale mismatch suggests B is standardised while CI is unstandardised; needs reconciliationR2-7Effect sizes / % changeDoneTable 6 addedR2-8Female stigma — cultural mechanismDonel.315; appropriately caveated as non-significantR2-9STI screening / unreliable sourcesDonel.295R2-10Hong Kong contextDonel.274R2-11Ethics approval timelinePartialExplained in response letter only; clarification not inserted into the manuscript's Ethics sectionR2-12Data availability per PLOS policyBorderlineSI route is acceptable but does not match the reviewer's request for a named repository; also ".scv" typo

Residual issues requiring action

Sample size inconsistency (not flagged previously, but visible). Methods (l.116) states "A total of 205 students participated," while Results (l.214) reports n=216. The 205 figure references a sample-size justification from another study; the actual analytical sample is 216. The Methods text should be corrected for internal consistency.

Reference attribution error. Methods l.116 cites "Al-Fadhli et al." for the Central India workshop study, but the reference list (Ref 21) is Machowska et al. The same study is cited correctly later (Ref 56). Author name must be corrected.

Table 4 The main issue is that the table seems to confuse regression coefficients with odds ratios, leading to mismatched confidence intervals and incoherent results. It likely reflects either a reporting error or a mislabeling of the statistical method.

Ethics timeline. The reviewer's clarification request should be reflected in the manuscript itself (Ethics section), not only in the response letter.

Light copy-edit pass to fix residual typos and the two reference-list artefacts (chatgpt.com utm strings, ResearchGate link).

Strengths to acknowledge

Genuinely novel contribution for the MENA region; first integration of HIV-KQ-18 and HPASS among medical students; appropriate validated instruments; honest treatment of limitations after revision; Table 6 substantially improves interpretability.

Bottom line for the editor: Accept after a focused minor revision addressing the five items above. None require new data collection or restructuring; all are correctable in a single revision cycle.

Reviewer #3: The authors have addressed a topical issue that seeks to identify the level of stigma against PLHIV among future care givers (medical students), and proffer solutions to promptly address the challenge. The manuscript is well written in simple English, the methods are well documented to allow for reproducibility, the statistical analyses are rigorous and thorough, the results have been well presented and the findings discussed. Limitations of the study have also been well documented. The authors have satisfactorily addressed all previous review comments.

**********

7. PLOS authors have the option to publish the peer review history of their article (what does this mean?). If published, this will include your full peer review and any attached files.

If you choose “no”, your identity will remain anonymous but your review may still be made public.

**Do you want your identity to be public for this peer review?** For information about this choice, including consent withdrawal, please see our Privacy Policy.

Reviewer #1: No

Reviewer #2: No

Reviewer #3: **Yes:** Oluwasanmi Akanji Adedokun

**********

Attachment Submitted filename: PONE-D-25-49448_R4_reviewer.pdf

# Editor report: Acceptance letter

**DOI:** 10.1371/journal.pone.0350810.r004

**Authors:** Hamufare Dumisani Mugauri

PONE-D-25-49448R1

PLOS One

Dear Dr. Hariz,

I'm pleased to inform you that your manuscript has been deemed suitable for publication in PLOS One. Congratulations! Your manuscript is now being handed over to our production team.

At this stage, our production department will prepare your paper for publication. This includes ensuring the following:

* All references, tables, and figures are properly cited

* All relevant supporting information is included in the manuscript submission,

* There are no issues that prevent the paper from being properly typeset

You will receive further instructions from the production team, including instructions on how to review your proof when it is ready. Please keep in mind that we are working through a large volume of accepted articles, so please give us a few days to review your paper and let you know the next and final steps.

Lastly, if your institution or institutions have a press office, please let them know about your upcoming paper now to help maximize its impact. If they'll be preparing press materials, please inform our press team within the next 48 hours. Your manuscript will remain under strict press embargo until 2 pm Eastern Time on the date of publication. For more information, please contact onepress@plos.org.

You will receive an invoice from PLOS for your publication fee after your manuscript has reached the completed accept phase. If you receive an email requesting payment before acceptance or for any other service, this may be a phishing scheme. Learn how to identify phishing emails and protect your accounts at https://explore.plos.org/phishing.

If we can help with anything else, please email us at customercare@plos.org.

Thank you for submitting your work to PLOS ONE and supporting open access.

Kind regards,

PLOS ONE Editorial Office Staff

on behalf of

Dr Hamufare Dumisani Mugauri

Academic Editor

PLOS One
